# Remodeling the pathological airway: advanced nanotechnology for diagnostics and therapeutics in cystic fibrosis

**DOI:** 10.1186/s12951-026-04689-4

**Published:** 2026-06-16

**Authors:** Ying Zou, Hang Zhao, Mengyi Feng, Zhaoqi Wu, Zhongping Cao, Zhonghao Fan, Martin Gluchman, Huazhe Yang, Chenyu Zhao, Peng Yu, Xudong Zhang

**Affiliations:** 1 Department of Rehabilitation Medicine, Shengjing Hospital of China Medical University, China Medical University, Shenyang, China; 2 Department of Respiratory Medicine, Shengjing Hospital of China Medical University, China Medical University, Shenyang, China; 3 Department of Hyperbaric Oxygen Medicine, The First Hospital of China Medical University, China Medical University, Shenyang, China; 4 Department of Neurosurgery, The First Hospital of China Medical University, China Medical University, Shenyang, China; 5 Department of China, School of Pharmacy, Medical University-The Queen’s University of Belfast Joint College, China Medical University, Shenyang, China; 6 School of Pharmacy, Queen’s University Belfast, Belfast, UK; 7 State Key Laboratory of Discovery and Utilization of Functional Components in Traditional Chinese Medicine, Shandong University, Jinan, China; 8 School of Intelligent Medicine, China Medical University, Shenyang, China

**Keywords:** Cystic fibrosis, Nanomedicine, Biosensor, Drug delivery system, Clinical translation

## Abstract

Cystic fibrosis (CF) constitutes a prototypical lethal genetic disorder characterized by profound pathophysiological barriers that severely compromise both diagnostic performance and targeted therapeutic efficacy. Although the advent of cystic fibrosis transmembrane conductance regulator (CFTR) modulators has revolutionized disease management, the critical clinical imperatives for ultra-sensitive diagnostic modalities and deep pulmonary delivery systems remain largely unmet. This comprehensive review critically evaluates the transformative potential of nanotechnology in remodeling the dual diagnostic and therapeutic landscapes of CF. Initially, we highlight the integration of advanced nanoscale sensors for the real-time and ultra-sensitive profiling of sweat biomarkers, specific genetic mutations, and respiratory metabolites. These innovations fundamentally elevate early diagnostic precision and continuous disease monitoring capabilities. Concurrently, we systematically elucidate how precision-engineered nanocarriers circumvent formidable mucosal and extracellular polymeric substance biofilms. These vehicles facilitate the targeted pulmonary delivery of diverse pharmacological payloads, including CFTR modulators, mucolytics, complex nucleic acid therapeutics and antimicrobial agents. Ultimately, transitioning from engineering conceptualization to clinical application, we dialectically analyze the multifaceted translational hurdles. This review establishes a rigorous roadmap for propelling diagnostic and therapeutic nanomedicine toward clinical accessible realities.

**Graphical abstract:**

**Figure:**
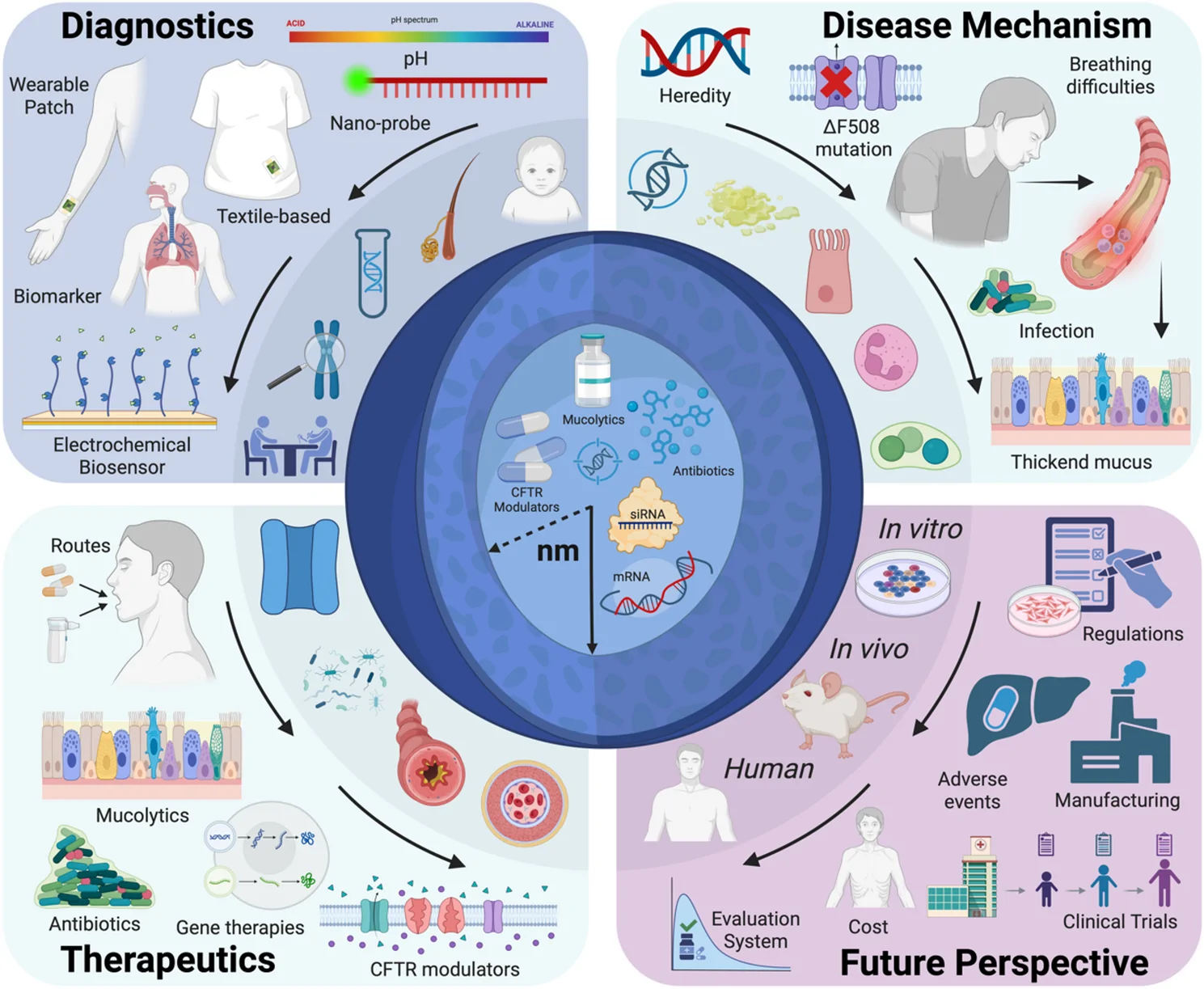

## Introduction

Cystic fibrosis (CF) is an autosomal recessive genetic disorder driven by mutations in the cystic fibrosis transmembrane conductance regulator (CFTR) gene [1]. It is the most prevalent lethal genetic disorder in Caucasian populations [2, 3]. Alterations in the CFTR gene yield a functionally defective CFTR protein, impairing the transmembrane transport of chloride and bicarbonate ions and destabilizing the ionic and fluid homeostasis across epithelial surfaces [4, 5]. This leads to depletion of the airway surface liquid and concentration of the periciliary fluid [6]. These events culminate in the condensation and accumulation of mucins, generating a highly viscous and tenacious mucus layer that is remarkably difficult to clear [7]. This aberrant mucus obstructs the airways and creates a favorable microenvironment for bacterial colonization and chronic infection [8]. The prolonged presence and biofilm formation of opportunistic pathogens, notably *Pseudomonas aeruginosa*, trigger a persistent and exaggerated inflammatory cascade [9–11]. Such sustained inflammation ultimately drives the structural degradation of the airway wall, the development of bronchiectasis, and a progressive decline in pulmonary function [12]. As pulmonary complications are the leading cause of mortality in CF patients, understanding this respiratory pathophysiology and its associated biological barriers is essential [13].

Current clinical management of CF continues to encounter substantial hurdles [14–16]. Diagnostically, the traditional sweat chloride test remains the gold standard [17]. However, the procedure is cumbersome and demands strict sample quality, restricting its utility for rapid point-of-care testing [18]. Genetic screening definitively identifies specific mutation profiles, but it is costly and slow, and commercial panels often fail to detect rare variants [19, 20]. Moreover, current diagnostic protocols rely on isolated parameters and cannot provide real-time, non-invasive monitoring of airway microenvironment dynamics. Therapeutically, the introduction of CFTR modulators has generated breakthrough clinical progress for individuals harboring specific genotypes [21]. Despite this success, a significant demographic of patients, particularly those with rare or nonsense mutations, still cannot benefit from these targeted interventions [22]. Even in treated patients, pre-existing structural lung damage and chronic infections are irreversible, requiring lifelong antibiotics, mucolytics, and anti-inflammatory agents to control disease progression [23–26]. Regarding pharmacological delivery, interventions administered via conventional oral or intravenous routes must negotiate the fundamental biological barrier of CF, specifically the highly concentrated and pathologically viscoelastic mucus layer [27]. CF mucus has a much smaller mesh pore size than healthy mucus, trapping conventional drug particles and resulting in subtherapeutic concentrations at target cells [28]. Attempts to overcome this poor pulmonary penetrance by escalating systemic dosages routinely trigger severe adverse effects, including nephrotoxicity and ototoxicity, which critically narrow the therapeutic window and undermine the viability of prolonged treatment regimens [29]. Therefore, developing nanomedicines capable of bypassing the CF mucus barrier for efficient pulmonary target accumulation is clinically imperative.

The convergence of nanoscale engineering and respiratory medicine represents a transformative shift from symptomatic management to dynamic microenvironmental modulation [30]. Conventional diagnostic modalities typically yield static physiological snapshots [31]. Conversely, nano-diagnostic platforms leverage unique plasmonic, magnetic, and electrochemical properties to enable the real-time interrogation of the pulmonary landscape [32]. These sophisticated biosensors bridge the diagnostic chasm between gross clinical phenotypes and underlying molecular pathology by detecting trace biomarkers with unprecedented sensitivity [33, 34]. On the therapeutic front, the hyperviscous CF airway acts as an impenetrable steric and electrostatic fortress [35]. Nanotechnology addresses this barrier by precisely tuning nanoparticle physicochemical properties [36]. By tuning particle size, shape, and surface charge, researchers can design delivery systems that evade muco-ciliary clearance [37]. Modifying these surfaces with hydrophilic polymers or biomimetic cellular membranes transforms passive drug receptacles into active mucosal navigators [38]. Furthermore, embedding these discrete nanoparticles within inhalable microscale matrices resolves a critical aerodynamic paradox. This specific hierarchical design ensures optimal deposition within the deep lung parenchyma while preserving the vital capacity for nanoscale tissue penetration upon localized release [39]. Consequently, these platforms orchestrate the spatiotemporal delivery of complex therapeutic regimens.

In light of this background, this review aims to systematically discuss recent research progress concerning the application of nanotechnology within the diagnostics and therapeutics of CF. This manuscript initially deconstructs the aberrant pulmonary pathophysiology that define the CF lung. Establishing this pathophysiological foundation is critical for the rational design of subsequent nano-therapeutic interventions. The narrative then critically evaluates emerging nano-diagnostic methodologies. We emphasize their progressive utility in quantifying genetic expressions, mapping localized ion fluctuations, and monitoring real-time physiological declines. Building upon this diagnostic framework, the review dissects the architectural strategies of diverse nanocarriers engineered to deliver distinct active pharmaceutical ingredients (API). We specifically analyze the encapsulation and targeted release of CFTR modulators, mucolytics, complex nucleic acid therapeutics and antimicrobial agents. To provide rigorous clinical context, we present an extensive comparative analysis weighing the pharmacokinetic advantages and inherent limitations of pulmonary inhalation against systemic administration routes. Finally, this review confronts the steep translational gradients existing between laboratory conceptualization and human clinical trials. We critically assess the regulatory complexities, scale-up manufacturing hurdles, and future developmental trajectories defining this specialized field. Ultimately, this work aims to construct a definitive strategic roadmap for clinicians and bioengineers, seeking to accelerate the translation of precision nanomedicine from theoretical constructs into tangible therapeutic realities for patients combating CF.

**Fig. 1:**
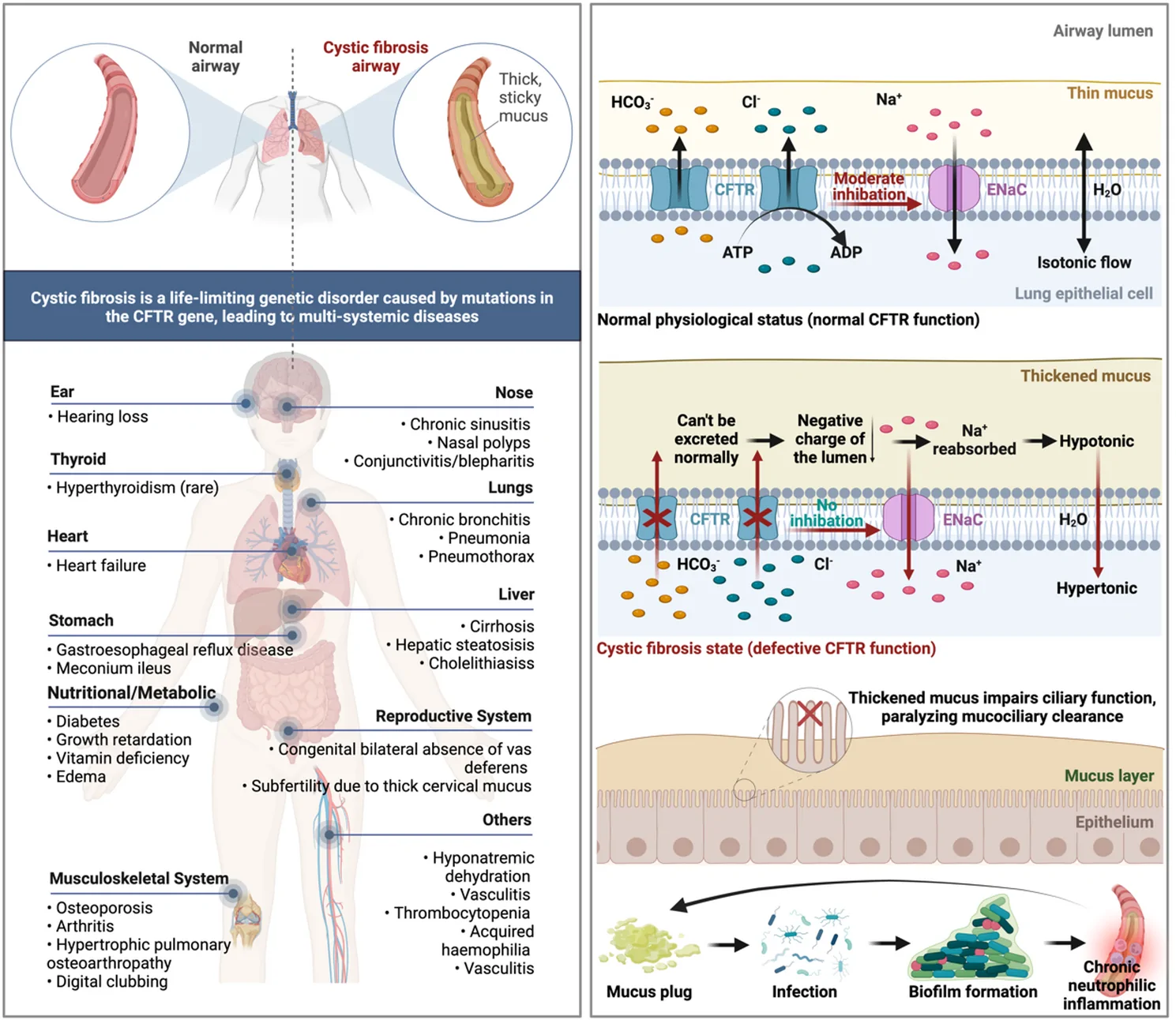
Integrated Pathophysiology of Cystic Fibrosis. This figure contrasts normal airway epithelial physiology with the core molecular defect of CFTR dysfunction, which drives airway ion and fluid transport imbalance, muco-ciliary clearance failure, infectious and inflammatory cascade, and the full range of multi-system clinical complications of this genetic disorder

## Pathophysiology of cystic fibrosis

CF is a life threatening autosomal recessive monogenic disorder primarily caused by pathogenic mutations in the CFTR gene located on the long arm of chromosome 7 [40]. Over 2000 distinct CFTR mutations have been identified to date [41]. The phenylalanine 508 deletion (ΔF508) remains the most prevalent global variant, predominant in Caucasian populations, and causes severe protein misfolding and premature degradation [42]. The CFTR gene encodes a unique ATP gated ion channel protein distributed extensively on the apical membrane of epithelial cells across multiple exocrine glands, including the respiratory tract, gastrointestinal tract, pancreas, and reproductive system [43]. It primarily regulates transmembrane chloride and bicarbonate transport (Fig. 1) [44, 45]. Within healthy absorptive respiratory epithelia, the surface CFTR channel works synergistically with the epithelial sodium channel (ENaC) to maintain intracellular and extracellular fluid and electrolyte homeostasis [46, 47]. Loss or dysfunction of CFTR due to genetic variants severely impairs chloride secretion into the airway lumen [48]. Concurrently, CFTR-mediated inhibition of ENaC is abolished.

This ion transport dysregulation drives excessive epithelial sodium absorption. Propelled by a strong osmotic gradient, massive amounts of water are forcibly reabsorbed into the intracellular space alongside sodium ions, triggering profound dehydration of the airway surface liquid layer. Depletion of the airway surface liquid collapses the periciliary layer, halting ciliary beating and paralyzing muco-ciliary clearance. Consequently, the dehydrated mucus becomes highly viscous, forming stubborn distal airway plugs [49–51]. This stagnant and hypoxic mucus microenvironment provides an exceptionally ideal niche for the extensive proliferation and colonization of various pathogenic microorganisms [52]. Consequently, patients become highly susceptible to recurrent bacterial infections, with chronic colonization by *Pseudomonas aeruginosa* and *Staphylococcus aureus* proving to be particularly lethal [53].

To clear this persistent microbial burden, the innate immune system becomes hyperactivated, recruiting large numbers of neutrophils to the respiratory tract [54]. This profound and sustained inflammatory response fails to eradicate the pathogens completely [55]. Instead, it causes widespread neutrophil necrosis [56]. These dying cells release high concentrations of proinflammatory cytokines such as IL-8, highly destructive neutrophil elastase, and an abundance of negatively charged extracellular DNA alongside free actin polymers [57]. These macromolecules crosslink in the airway lumen, markedly increasing sputum viscoelasticity and rigidity [58]. This biochemical alteration establishes a vicious cycle in the lungs interwoven with mucus obstruction, refractory chronic infection, intense inflammation, and irreversible tissue damage [59]. As the disease progresses, this unrelenting deep inflammation and infection irreversibly destroy the airway wall architecture, inducing severe bronchiectasis, pulmonary parenchymal fibrotic scarring, and progressive failure of pulmonary ventilation and gas exchange [60]. This structural destruction is the hallmark pathological feature of CF and the leading cause of morbidity and mortality in affected patients.

Beyond the respiratory tract, CF pathology impairs exocrine function in multiple organ systems. In the gastrointestinal and digestive systems, highly abnormal and viscous secretions physically obstruct the pancreatic ducts, hindering the smooth discharge of key digestive enzymes like trypsin into the small intestine. Over time, this obstruction leads to the autodigestion and destruction of pancreatic exocrine tissue, ultimately triggering severe pancreatic exocrine insufficiency, malabsorption of fats and fat soluble vitamins, and progressive malnutrition manifestations such as stunted growth [61]. Pancreatic parenchymal fibrosis also damages islet endocrine cells, causing CF-related diabetes, which shares features of both type 1 and type 2 diabetes [62]. In the hepatobiliary system, thickened biliary secretions cause cholestasis and focal biliary cirrhosis, which may progress to portal hypertension in advanced disease. Likewise, in the reproductive system, the abnormal concentration of secretions leads to congenital bilateral absence of the vas deferens in almost all male patients during embryonic development or postnatally, subsequently causing highly prevalent infertility [63].

## Application of nanotechnology in the diagnostics of cystic fibrosis

### Challenges in clinical diagnosis

Current clinical diagnostic protocols strictly adhere to global consensus guidelines, establishing newborn screening, sweat chloride quantification, and genetic mutation profiling as the functional and molecular gold standards [16, 64, 65]. However, transitioning these established methods to point-of-care settings faces significant analytical and operational limitations [66, 67].

Conventional pilocarpine iontophoresis, while standard, is operationally cumbersome, relies on benchtop infrastructure, and suffers from delayed analytical turnarounds[68]. Open fluid collection techniques frequently result in sample leakage and insufficient yields within neonatal and infant cohorts. This methodology completely precludes in situ real time surveillance and demonstrates insufficient sensitivity for resolving borderline clinical presentations, which inevitably exacerbates the risk of diagnostic omission or misclassification [64]. In the realm of molecular confirmation, standard genetic assays are hindered by protracted nucleic acid hybridization workflows that routinely consume three to twelve hours per run [69]. These platforms exhibit constrained analytical sensitivity when differentiating single nucleotide polymorphisms and minute pathogenic deletions [70]. Such constraints render them incompatible with rapid point-of-care requirements and highlight critical limitations regarding the detection thresholds for trace genomic samples [71–74]. Similarly, the evaluation of respiratory biomarkers necessary for prognostic profiling mandates invasive large volume sampling procedures [75]. Such interventions inherently disrupt the delicate physiological equilibrium of the pulmonary mucosa [76]. Traditional analytical techniques demonstrate poor resistance to biological interference and fail to provide precise quantification of ultra-trace targets or gaseous inflammatory mediators through nondestructive in situ monitoring [77].

These inherent clinical bottlenecks severely restrict the capacity for ultra-early screening, precise diagnostic confirmation, and dynamic longitudinal monitoring of CF [78]. The profound integration of functional nanomaterials with highly sensitive biosensing technologies now offers a groundbreaking technical advance capable of overcoming these fundamental clinical limitations.

**Fig. 2:**
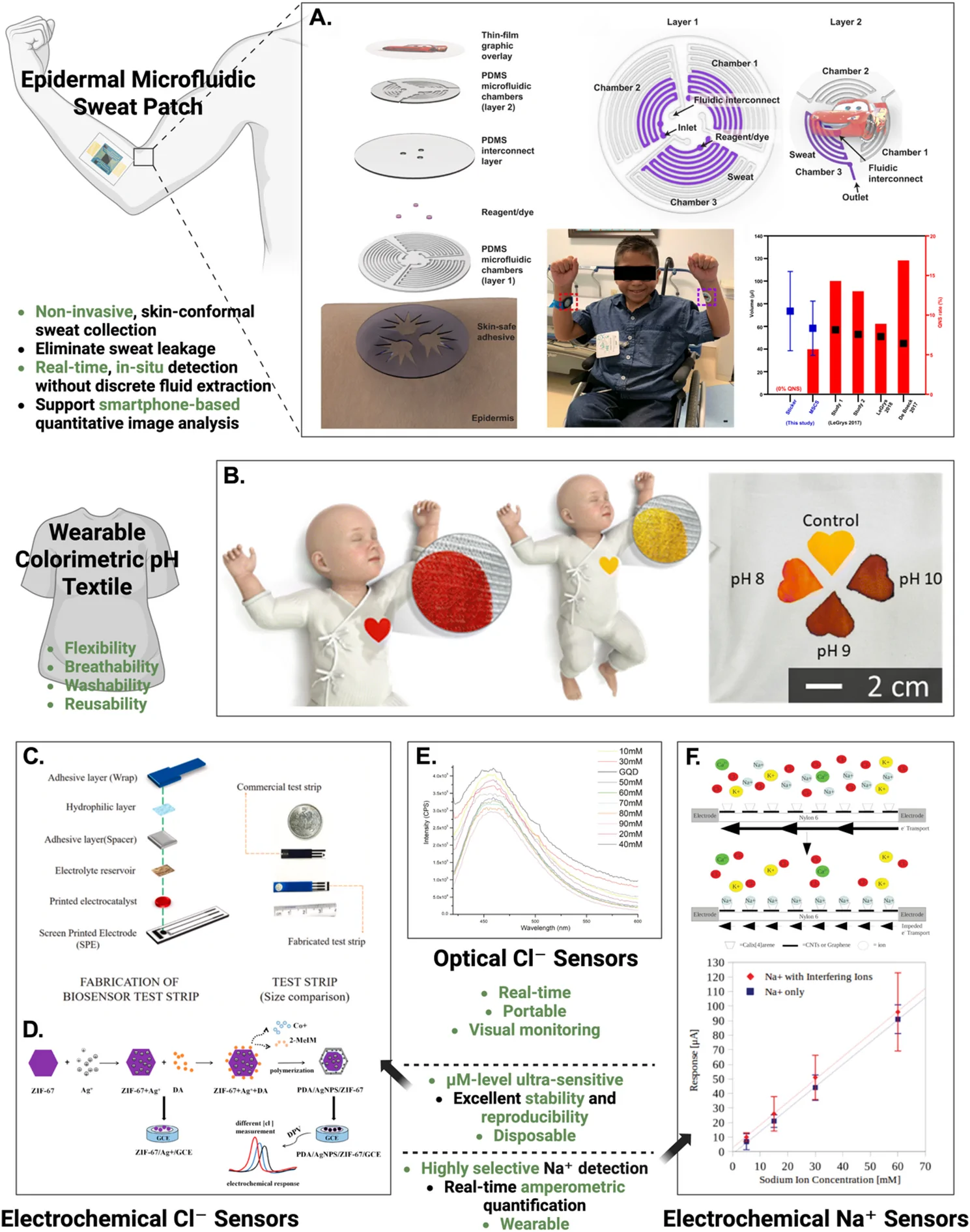
Overview of Advanced Sweat-based Sensing Platforms for Electrolyte Biomarker Detection in the Diagnostics and Management of Cystic Fibrosis (CF). **(A)** Schematic and application demonstration of an epidermal microfluidic sweat patch for CF diagnosis. The panel illustrates the multilayer polydimethylsiloxane (PDMS) microfluidic construction of the patch, which enables non-invasive, skin-conformal sweat collection with minimal leakage risk. This platform supports real-time, in-situ colorimetric detection of sweat chloride without discrete fluid extraction, as well as smartphone-based quantitative image analysis [79]. **(B)** Wearable colorimetric textile sensor for sweat pH monitoring in CF patients. The panel shows the integration of curcumin-incorporated thermoplastic polyurethane (C-TPU) electrospun fiber sensors into infant swaddling and patient clothing, with a visual demonstration of colorimetric response to varying pH conditions. This textile-based sensor features high flexibility, breathability, washability and reusability, and provides direct visual readout of sweat pH changes associated with CF exacerbation [80]. **(C)** Layered fabrication scheme and physical prototype of a disposable electrochemical biosensor test strip for sweat chloride detection. The panel details the assembly of functional layers including an adhesive wrap, hydrophilic layer, spacer layer, electrolyte reservoir, printed electrocatalyst and screen printed electrode, alongside a size comparison between the fabricated test strip and a commercial test strip [81]. **(D)** Electrochemical chloride sensor based on zeolitic imidazolate framework-67/silver nanoparticles/polydopamine (ZIF-67/AgNPs/PDA) nanocomposites. The panel depicts the synthetic route of the nanocomposite material and the differential pulse voltammetry (DPV)-based chloride sensing mechanism. This sensor delivers high response reproducibility and stability for sweat chloride quantification in CF diagnosis [82]. **(E)** Optical chloride sensor based on graphene quantum dots (GQDs) for CF-related sweat analysis. The panel presents the fluorescence emission spectra of GQDs with increasing chloride concentrations and highlights the core advantages of this sensing platform including real-time detection, portability, visual monitoring capability, micromolar-level sensitivity, excellent stability and disposability[83]. **(F)** Electrochemical sodium ion sensor for sweat sample quantification. The panel illustrates the selective sodium ion recognition mechanism at the multiwall carbon nanotube (MWNT)/calixarene functionalized nylon-6 mat interface, and the amperometric response calibration curves for sodium ion detection with and without common interfering ions in sweat. This platform enables real-time amperometric quantification of sodium ions with high selectivity for wearable sweat analysis [84]. Reproduced with permission

### Wearable and electrochemical sweat profiling

The profound integration of flexible microfluidic architectures with functional nanomaterials has recently yielded innovative methodologies for the noninvasive, in situ, and real time interrogation of eccrine sweat. Tyler R. Ray et al. utilizing soft lithography fabrication techniques have engineered an epidermal microfluidic sweat sticker that achieves perfect conformal adhesion to the skin (Fig. 2A) [79]. The internal configuration of this device incorporates microscale fluidic channels coupled with nanoscale sealing interfaces, effectively eradicating fluid leakage. This structural integrity facilitates the real time quantitative image analysis of chloride sensitive colorimetric reagents via smartphone cameras, thereby attaining clinical grade diagnostic accuracy without the necessity of discrete fluid extraction. Regarding wearable pH surveillance, electrospun intelligent textiles formulated from curcumin and thermoplastic polyurethane provide a robust alternative to fragile and unwieldy mechanical pH probes (Fig. 2B) [80]. This matrix exploits the extraordinary specific surface area and rapid mass transfer kinetics inherent to nanofibers. Consequently, the curcumin molecules undergo a measurable structural transition by enol-keto tautomerization in response to pH fluctuations, yielding a prominent visual colorimetric shift. The hierarchical porous architecture of the nanofibrous membrane endows the sensor with exceptional breathability and biocompatibility while maintaining rigorous structural resilience against neutral washing protocols to ensure extended reusability.

For the optical quantification of chloride ions, amorphous graphene quantum dots with diameters between 12 and 15 nm demonstrate exceptional optical characteristics [83]. Exploiting the specific fluorescence quenching effect exerted by chloride ions on these quantum dots yields a robust linear correlation characterized by an R^2^ value of 0.98 across a 100 mM dynamic range, achieving a limit of detection approaching 10 mM (Fig. 2E).

While colorimetric and optical strategies significantly optimize patient compliance, electrochemical nanosensors present an unparalleled advantage concerning ultra-trace analytical sensitivity. To precisely quantify sodium ions, highly conductive amperometric real time ion sensors have been fabricated by functionalizing multiwalled carbon nanotubes with selectively complexing calixarene macromolecules and depositing this composite onto flexible nylon 6 substrates (Fig. 2F) [84]. Within the critical domain of chloride sensing, novel nanocomposite architectures continuously redefine the absolute boundaries of detection limits for CF (Fig. 2D) [82]. Specifically, porous yolk shell metal organic framework composites utilizing a polydopamine coating to stabilize internal silver nanoparticles ensure excellent response reproducibility and depress the chloride limit of detection down to 1 mM.

In a profoundly disruptive approach, disposable screen printed test strips functionalized with silver nanoparticle decorated reduced graphene oxide leverage the immense specific surface area of two dimensional graphene alongside the reversible silver and silver chloride redox mechanism (Fig. 2C) [81]. This platform achieves an extraordinary analytical sensitivity of 165.2 ± 4.3 µA/mM/cm^2^, driving the limit of detection to an unprecedented 2.46 µM. It exhibits a relative standard deviation of merely 2.63% across repetitive testing cycles and three months of storage, completely fulfilling the rigorous accuracy and stability criteria required for point-of-care testing. Furthermore, inducing the self-assembly and aggregation of gold nanorods directly on aluminum interdigitated electrodes via the sodium chloride sample itself enhances the quantitative current signal performance by 87.92% when compared to systems utilizing statically immobilized nanorods[85].

Collectively, the convergence of flexible microfluidics and diverse nanomaterials establishes a formidable hardware foundation for the noninvasive characterization of CF, effectively shattering traditional sensitivity ceilings (Table 1). However, despite these promising analytical capabilities, the majority of current sweat nanosensors remain confined to the proof of concept stage utilizing artificial sweat. While certain advanced microfluidic patches have undergone small scale patient cohort testing against standard pilocarpine iontophoresis, widespread clinical deployment remains premature. To bridge this critical translational gap, future engineering efforts must actively resolve principal barriers, which include severe sensor fouling by skin lipids, baseline electrical drift requiring continuous calibration, and uncertain long term adhesive biocompatibility.

**Fig. 3:**
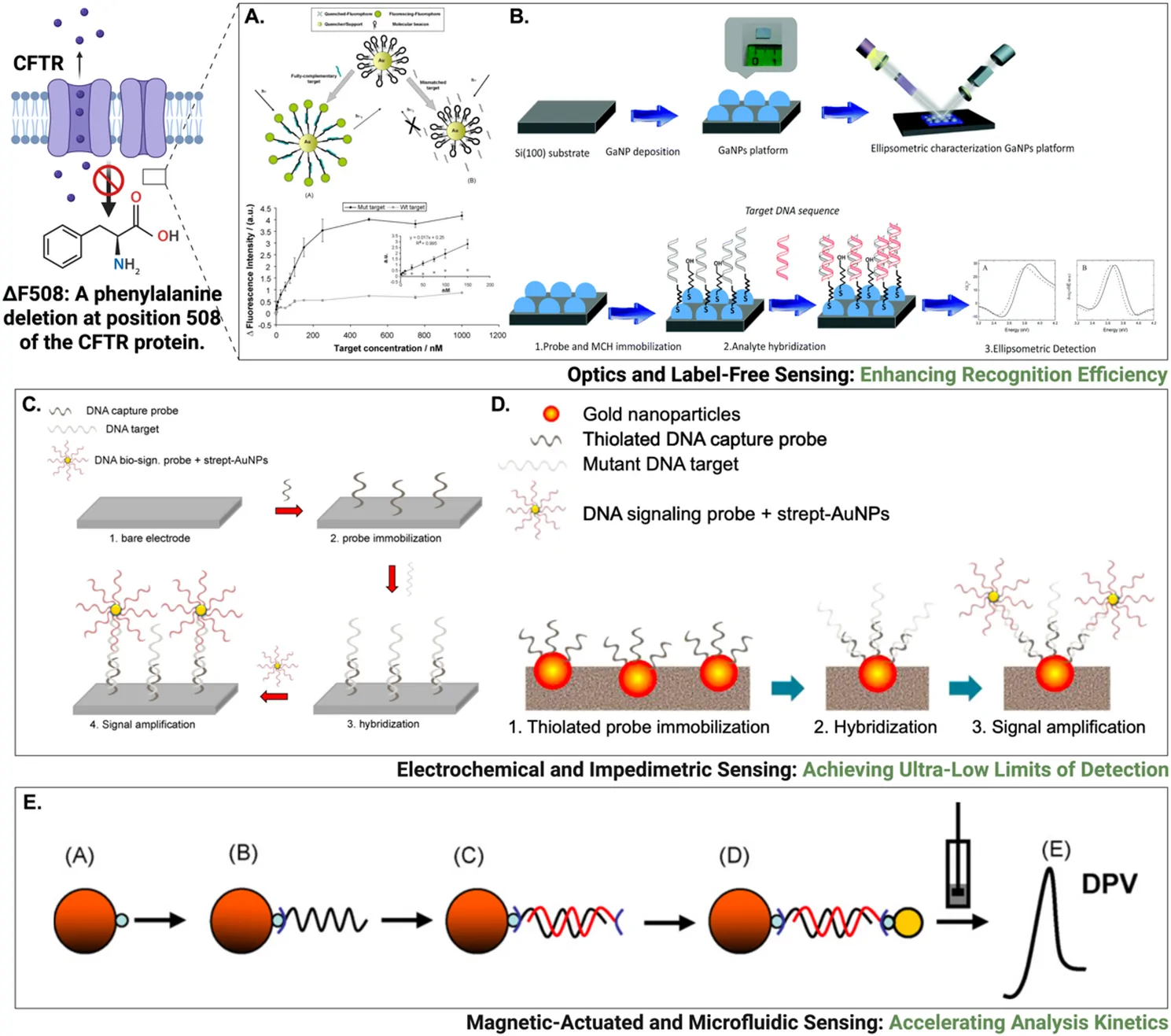
Overview of Advanced Biosensing Platforms for Detecting Cystic Ribrosis (CF)-associated Mutations in the Cystic Fibrosis Transmembrane Conductance Regulator (CFTR) Gene. The left schematic illustrates the core pathological basis of CF, in which deletion of phenylalanine at position 508 (ΔF508) of the CFTR protein results in impaired protein function and dysregulated transmembrane ion transport. **(A)** Working principle and quantitative performance of a gold nanoparticle-based fluorescent molecular beacon system for specific detection of the CF-related ΔF508 mutation, including the fluorescence response profile across the tested target DNA concentration range [86]. **(B)** Label-free ellipsometric sensing platform based on gallium plasmonic nanoparticles (GaNPs) for CFTR gene mutation detection, showing the GaNP sensing substrate fabrication workflow, DNA hybridization and detection procedure, and representative ellipsometric characterization results [87]. **(C)** Impedimetric genosensing protocol using multi-walled carbon nanotube-modified screen-printed electrodes for CF-associated DNA polymorphism detection, covering sequential steps of bare electrode preparation, capture probe immobilization, target DNA hybridization, and signal amplification with streptavidin-modified gold nanoparticles [88]. **(D)** Impedimetric sensing strategy using gold nanoparticle-integrated graphite-epoxy nanocomposite electrodes for ultra-sensitive detection of the CF-linked triple-base deletion mutation, with key steps of thiolated DNA probe immobilization on gold nanoislands, target sequence hybridization, and terminal signal amplification [89]. **(E)** Magnetically actuated electrochemical genosensing approach with differential pulse voltammetry (DPV) readout for rapid detection of CF-related DNA targets, outlining the full workflow from capture probe conjugation to paramagnetic beads, DNA hybridization, gold nanoparticle tagging, and final magnetically triggered electrochemical detection [90]. Reproduced with permission

### Point-of-care genosensing and target amplification

The definitive diagnosis of CF hinges on the precise detection of CFTR gene mutations, notably the prevalent ΔF508 variant. Conventional genetic hybridization techniques remain hampered by protracted assay durations and restricted analytical sensitivity. Leveraging their extraordinary specific surface areas and distinctive optoelectronic properties, nanomaterials have catalyzed profound advancements in the development of ultra-sensitive point-of-care genosensors.

Within the optical sensing domain, nanomaterials substantially optimize probe performance. Valerio Beni et al. have precisely anchored a 31 nucleotide fluorescent molecular beacon onto 13 nm gold nanoparticles via a thiol linker. This platform achieves clear differentiation between mutant and wild type CF sequences at target concentrations as low as 1 nM (Fig. 3A) [86]. To further streamline operational protocols, Antonio García Marín et al. have utilized gallium plasmonic nanoparticles deposited onto silicon substrates (Fig. 3B) [87]. Capitalizing on a pseudodielectric function that is acutely responsive to perturbations in the surrounding surface dielectric medium, this configuration successfully executes the label free detection of specific mutations within large genomic DNA isolated from blood cells. Crucially, this methodology completely circumvents the necessity of introducing hybridization suppressors, such as formamide, into the analytical solution.

Electrochemical nanocomposites demonstrate an overwhelming advantage in depressing sensitivity thresholds. By integrating multiwalled carbon nanotubes with streptavidin modified gold nanoparticles for silver enhanced signal amplification, impedimetric genosensors achieve a limit of detection of approximately 100 pM for the three base pair deletion sequence (Fig. 3E) [90]. When electrode architectures are further upgraded to incorporate gold nanoisland graphite epoxy composite materials, rapid and stable thiol immobilization drastically mitigates steric hindrance among the DNA probes. This structural refinement drives the limit of detection down to an astonishing 2.25 fM (Fig. 3D) [89]. Furthermore, the application of electrodeposition and stripping techniques utilizing sacrificial copper nanostructures enables the random nanopatterning of probes on electrode surfaces, establishing an exceptional foundational matrix for amperometric genosensors [91].

The sluggish hybridization kinetics caused by the free diffusion of target molecules represent a critical vulnerability of conventional assays. Genomagnetic sensors employing a sandwich format, which combines paramagnetic beads with gold nanoparticle modified probes, facilitate highly efficient electrochemical quantification and robust single base mismatch discrimination (Fig. 3C) [88]. In an even more disruptive approach, systems utilizing spin valve sensors and alternating current magnetic fields generated by U shaped current lines actively focus magnetically labeled DNA targets directly onto the sensing domains. This magnetic enrichment effect precipitously abbreviates the conventional 3 to 12 h hybridization requirement down to a mere 15 to 30 min while simultaneously permitting the real-time surveillance of binding kinetics [92].

Comparative analysis across these modalities reveals that optical plasmonic nano-probes have fundamentally revolutionized specific recognition strategies (Table 1). Electrochemical platforms driven by graphene and gold nano-islands propel analytical sensitivity into the fM regimen, whereas magnetic nanoparticles completely eradicate the temporal delays associated with passive diffusion. While the synergistic implementation of these nanoscale technologies establishes a formidable foundation for ultra-sensitive point-of-care screening, their clinical realism demands critical appraisal. Currently, these platforms operate predominantly at the preclinical prototype stage, validating analytical performance on synthetic oligonucleotides or purified DNA rather than raw clinical samples. When compared against standard polymerase chain reaction techniques, their principal translational barriers remain the absence of automated on chip nucleic acid extraction and limited multiplexing capacities for rare mutations.

**Fig. 4:**
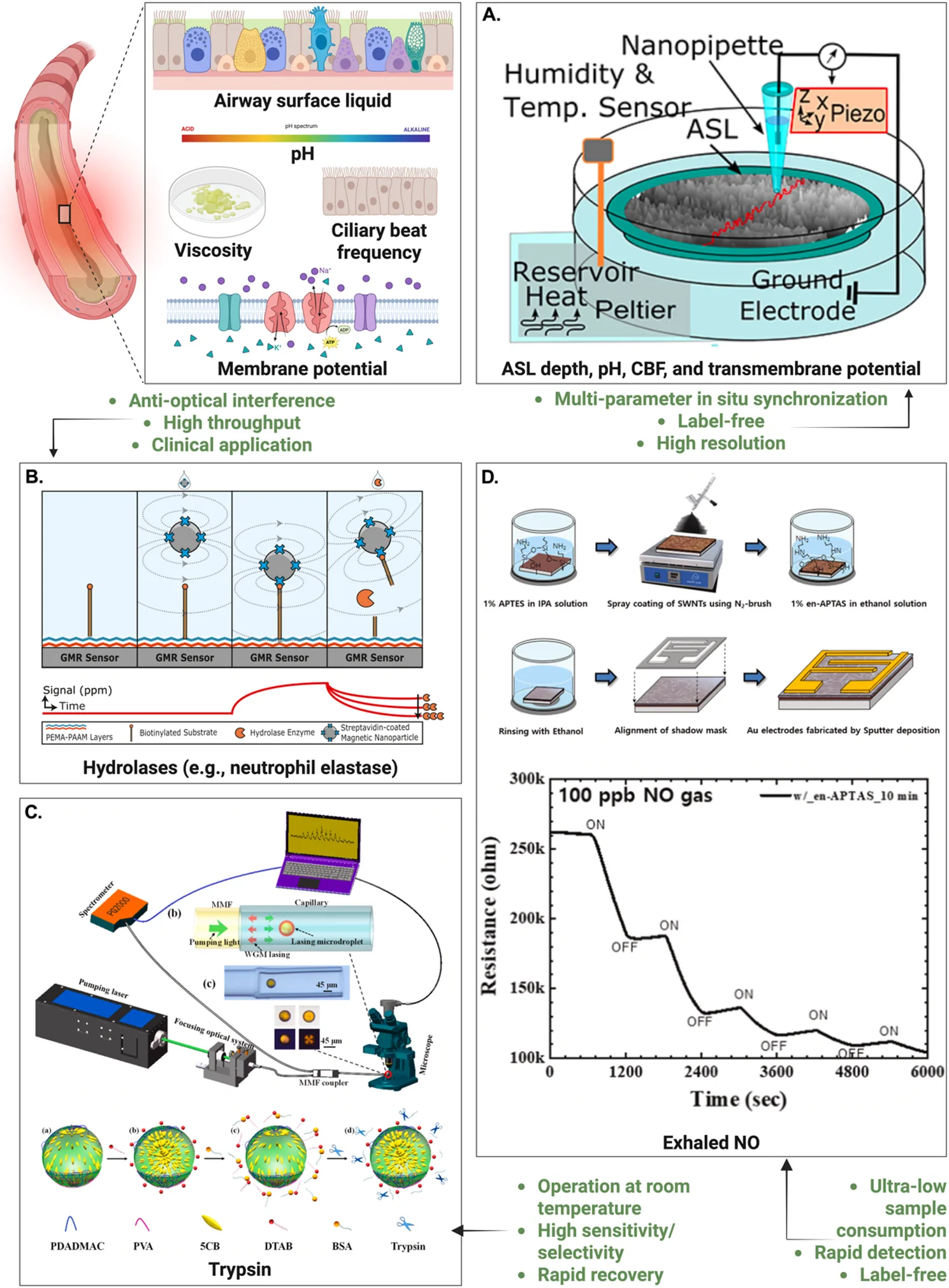
Nanosensor Platforms for Detection of Cystic Fibrosis (CF)-related Pathophysiological Biomarkers. **(A)** Scanning ion conductance microscopy-based nanosensor for label-free, synchronous measurement of airway surface liquid properties and epithelial cell function, featuring nanoscale resolution and non-perturbative assessment of native sample phenotypes [93]. **(B)** Giant magnetoresistive spin valve sensor array for real-time, wash-free quantification of hydrolase activity, enabling multiplexed detection of key biomarkers such as neutrophil elastase in CF sputum specimens [94]. **(C)** Integrated fiber sensor based on the whispering gallery mode of liquid crystal microdroplets for rapid trypsin detection, with ultra-low sample consumption, high specificity in human serum, and a response time of less than 200 s [95]. **(D)** Defect-functionalized single-walled carbon nanotube nitric oxide gas sensor, which achieves highly sensitive and selective detection of ppb-level NO at room temperature for non-invasive exhaled breath analysis in CF and other inflammatory airway diseases [96]. Reproduced with permission

### Real-time respiratory and metabolic monitoring

The pathology of CF is not exclusively manifested in genetic mutations and sweat electrolyte abnormalities. The direct cause of mortality frequently stems from the deterioration of the respiratory microenvironment, such as the hyperviscosity of airway surface liquid, and the subsequent chronic inflammation. Conventional assays for respiratory biomarkers typically necessitate large volume sampling, centrifugal extraction, or the use of susceptible fluorescent labels, all of which are highly prone to disrupting the fragile physiological milieu. The integration of nanotechnology and miniaturized sensors has propelled respiratory pathological assessment into a new era characterized by nondestructive in situ analysis, ultra-low sample consumption, and robust interference resistance.

A delicate equilibrium encompassing the rheology, pH, and depth of the airway surface liquid is critical for pulmonary defense mechanisms. Traditional measurement methodologies severely perturb this vulnerable system. To circumvent this diagnostic challenge, investigators have developed a suite of nanosensor probes mounted onto a scanning ion conductance microscope (Fig. 4A)[93]. These nanoscale probes facilitate, for the first time, the near simultaneous quantification of the complex physicochemical properties of the airway surface liquid and epithelial cell function. This is achieved in a completely label free manner without sample perturbation, thereby providing invaluable in situ data for elucidating the pathophysiology of CF airways.

Respiratory inflammation and nutritional deficiencies culminate in aberrant activities of specific hydrolases, including neutrophil elastase and trypsin. Conventional fluorometric assays used to quantify enzyme activity in the sputum of CF patients are frequently compromised by autofluorescence and photobleaching inherent to complex biofluids. Consequently, a novel sensor based on giant magnetoresistive spin valves has been proposed (Fig. 4B) [94]. This platform conjugates hydrolase substrates with magnetic nanoparticles. Upon substrate cleavage by sputum enzymes, the magnetic nanoparticles detach and induce a highly precise alteration in electrical resistance. This mechanism not only enables wash free, real time data acquisition but also supports high throughput multiplexed detection via an array of 80 independently addressable sensors, perfectly circumventing optical interference. Regarding ultra-trace detection, a compact optical fiber sensor integrating the amplification capability of liquid crystals with the high sensitivity of whispering gallery modes has demonstrated extraordinary efficiency (Fig. 4C) [95]. Targeting abnormal trypsin levels, this system compresses the response time to under 200 s and reduces sample consumption to an unprecedented 3 nL. It exhibits excellent recovery rates ranging from 97% to 112% in human serum tests, establishing the feasibility of point-of-care testing using minute volumes of exhaled breath condensate or capillary blood.

Exhaled nitric oxide is a pivotal noninvasive gaseous biomarker for respiratory inflammation. Achieving precise parts per billion level detection at room temperature presents a significant analytical challenge. A cost-effective semiconducting gas sensor has been successfully fabricated by forming a network of single walled carbon nanotubes between gold electrodes and functionalizing them with en-APTAS (Fig. 4D) [96]. Coupled with an optimized rinsing protocol that elevates the recovery rate to 90.34%, this sensor generates a significant response of 28.64% to 100 parts per billion of nitric oxide gas at room temperature. It concurrently demonstrates profound selectivity and interference resistance against carbon monoxide and volatile organic compounds.

In summary, through mechanisms encompassing magnetoresistance, optical resonance, and carbon nanotube interfaces, these advanced technologies provide a non-destructive, high-fidelity snapshot of the CF respiratory microenvironment (Table 1). While they successfully elevate the detection of metabolic biomarkers to exceptional levels of sensitivity and interference resistance, their clinical realism demands critical appraisal. Currently, respiratory nanosensors reside predominantly at the analytical demonstration stage, relying on simulated breath condensates or ex vivo sputum with severely limited sample sizes. When evaluated against conventional mass spectrometry or immunoassays, these prototypes face significant translational barriers. The extreme hyperviscosity of authentic pathological sputum frequently induces rapid sensor degradation, and translating trace gaseous variations into actionable clinical decisions still lacks established regulatory baselines.

**Table 1:** Critical Summary of Advanced Nanoscale Diagnostic Platforms for Cystic Fibrosis

Target Biomarker	Nanoplatform Architecture	Detection Modality	Analytical Sensitivity (LOD)	Translational Stage and Principal Barriers
Sweat Chloride [79]	Epidermal microfluidic patch integrated with colorimetric reagents	Optical (Smartphone imaging)	Clinical diagnostic range	Small-scale patient cohort. Principal barriers include severe sensor fouling by skin lipids and uncertain long-term adhesive biocompatibility during prolonged wear.
Sweat Chloride [82]	ZIF-67/Ag NPs/polydopamine yolk-shell nanocomposites	Electrochemical (Differential pulse voltammetry)	1 mM	Proof-of-concept. Validated solely in artificial sweat. Future translation requires continuous calibration against baseline electrical drift in authentic biofluids.
CFTR Mutation (ΔF508) [89]	Gold nanoislands graphite-epoxy composite	Electrochemical (Impedimetric genosensing)	2.25 fM	Preclinical prototype. Detection relies exclusively on purified oligonucleotides, severely lacking integrated automated on-chip nucleic acid extraction capabilities.
CFTR Target Genes [92]	Magnetic spin-valve sensors coupled with U-shaped current lines	Magnetoresistive (AC field focusing)	< 83 fmol/µL	Analytical demonstration. Despite compressing hybridization time to 15 min, inter-sensor signal deviations up to 50% and complex hardware integration impede point-of-care deployment.
Airway Surface Liquid [93]	Nanoscale probes mounted on a scanning ion conductance microscope	Topographical and electrochemical profiling	Sub-micrometer spatiotemporal resolution	Ex vivo cell culture stage. The highly invasive operational setup and reliance on sophisticated benchtop infrastructure preclude in vivo real-time patient surveillance.
Sputum Trypsin [95]	Liquid crystal microdroplets integrated with compact optical fibers	Optical (Whispering gallery mode)	0.003 mg/mL (using 3 nL samples)	Analytical demonstration. The extreme spatiotemporal heterogeneity and hyperviscosity of authentic pathological sputum induce rapid sensor degradation and optical interference.
Exhaled Nitric Oxide [96]	Defect-functionalized single-walled carbon nanotube network with en-APTAS	Semiconducting gas sensing	< 100 ppb at room temperature	Preclinical prototype. Translating these trace parts-per-billion gaseous variations into actionable clinical decisions currently lacks established regulatory and physiological baselines.

This table critically evaluates the developmental stage and translational barriers of emerging diagnostic nanoplatforms. While nanoscale architectures confer exceptional analytical sensitivity and minimize sample consumption, their progression to regulatory readiness is frequently impeded by the complex physicochemical nature of cystic fibrosis biofluids. Transitioning these prototypes to point-of-care clinical deployment strictly mandates further validation utilizing authentic patient-derived samples and integrated sample preparation modalities. Abbreviations: CFTR, cystic fibrosis transmembrane conductance regulator; Ag NPs, silver nanoparticles; ZIF, zeolitic imidazolate framework; AC, alternating current

## Application of nanotechnology in the treatment of cystic fibrosis

### Challenges in therapeutics

#### Physicochemical deficits of current drugs

CF pharmacotherapy for pulmonary complications follows three core goals: restoring CFTR function, controlling chronic airway infection, and degrading accumulated viscous mucus (Table 2) [97, 98]. In clinical practice, these strategies depend on specific representative agents. Triple CFTR modulators, exemplified by elexacaftor/tezacaftor/ivacaftor, target the fundamental restoration of channel ion transport capabilities [99, 100]. Inhaled or systemic antibiotics (tobramycin, aztreonam) treat chronic *Pseudomonas aeruginosa* infection [101]. Concurrently, dornase alfa and hypertonic saline operate as standard mucolytics and osmotic regulators to disrupt DNA crosslinked networks and promote the physical expectoration of sputum [102, 103].

Despite achieving significant clinical milestones in improving forced expiratory volume in one second and reducing pulmonary exacerbation frequencies, these conventional therapeutics exhibit profound limitations rooted in their physicochemical and biochemical profiles [104]. Primary concerns arise with CFTR modulators like ivacaftor, which are predominantly highly hydrophobic small molecules possessing aqueous solubilities typically below 0.05 µg/mL [105]. Their oral bioavailability remains highly erratic and strictly dependent on concomitant high fat dietary intake [106]. Long-term ivacaftor treatment destabilizes membrane-rescued ΔF508-CFTR and accelerates its degradation [107].

Secondary challenges involve the chemical inactivation of free inhaled antibiotics within the pathological microenvironment. Cationic tobramycin binds anionic mucins and high-molecular-weight DNA in CF sputum via electrostatic interactions [108]. This interaction, compounded by low pH and elevated divalent cation concentrations, precipitates a substantial loss of bactericidal activity [109]. By contrast, aztreonam has a short half-life (~2.1 h) and poor reconstituted stability, hampering sustained therapeutic drug levels [110].

Macromolecular biologics such as dornase alfa demonstrate extreme sensitivity to the G-actin abundantly released in CF sputum during mucolytic action [111]. This profound biochemical inhibition features a binding inhibition constant of approximately 1 nM and significantly attenuates the catalytic efficacy required for DNA degradation [112]. Pancreatic enzyme replacement therapy, utilized to ameliorate digestive deficits, is highly susceptible to inactivation by gastric acid and pepsin [113]. While enteric coating technologies are routinely applied, inadequate bicarbonate secretion in the duodenum of CF patients creates an abnormally acidic local environment where the pH generally falls below 4. Low pH inhibits coat dissolution and blocks targeted intestinal enzyme release.

Regardless of oral, intravenous or inhaled administration, all conventional drugs face a complex CF pathological microenvironment. A multitude of overlapping biological defense barriers span anatomical, physical, chemical, and cellular dimensions. These encompass high mucus impedance in the gastrointestinal tract, aberrant systemic metabolic clearance rates, pulmonary vascular endothelial restrictions, dense pathological mucus meshes, recalcitrant bacterial biofilms, and rigorous macrophage mediated phagocytic clearance mechanisms. As a result, free drug concentrations drop exponentially before reaching lesional sites. These multifaceted barriers severely limit current pharmacotherapy, creating an urgent need for nanotechnology to revolutionize drug delivery.

**Table 2:** Comprehensive Overview of Marketed Therapeutics for Cystic Fibrosis: Mechanisms, Delivery Barriers, and Adverse Profiles

Drug Name	Class	Simplified Mechanism of Action	Physicochemical and Delivery Limitations	Rationale for Nanotechnology Intervention
Elexacaftor/Tezacaftor/Ivacaftor (Trikafta/Kaftrio)	CFTR Modulator (Triple Therapy)	Corrects misfolding of the mutant protein and increases the open probability of CFTR channels on the cell surface.	Highly hydrophobic drug molecules; oral absorption is heavily dependent on high-fat meals, making it difficult to maintain stable and effective blood drug concentrations in vivo.	Inhalable lipid nanocarriers bypass gastrointestinal absorption and dietary fat dependence, enabling direct pulmonary delivery.
Lumacaftor/Ivacaftor (Orkambi)	CFTR Modulator (Dual Therapy)	Targets the ΔF508 mutation to correct protein trafficking defects and enhance channel gating function.	Lumacaftor is a strong CYP3A inducer, leading to extensive drug-drug interactions; long-term use significantly alters plasma concentrations of lipid-soluble vitamins (A and E).	Polymeric nanoparticles concentrate drugs locally in the epithelium, circumventing hepatic metabolism and systemic drug interactions.
Tezacaftor/Ivacaftor (Symdeko)	CFTR Modulator (Dual Therapy)	Improves CFTR trafficking and channel opening for ΔF508 and certain residual function mutations.	Also requires co-administration with fat-containing foods; extensively metabolized by hepatic CYP3A, presenting complex pharmacokinetic profiles.	Mucopenetrating PEGylated nanocarriers facilitate targeted intracellular delivery, stabilizing pharmacokinetics independent of systemic metabolism.
Ivacaftor (Kalydeco)	CFTR Modulator (Monotherapy)	Acts as a potentiator, increasing the open probability of existing CFTR channels at the cell membrane (targeting gating mutations).	Poor aqueous solubility; systemic administration lacks tissue distribution specificity, leading to non-target organ exposure.	Mesoporous silica nanoparticles ensure localized, controlled release at the apical epithelium, minimizing systemic off-target exposure.
Tobramycin (TOBI, TOBI Podhaler)	Inhaled Antibiotic	Aminoglycoside; binds to the bacterial 30 S ribosomal subunit to inhibit protein synthesis.	Cationic nature makes it highly susceptible to inactivation by binding to negatively charged mucins and DNA in sputum; free molecules struggle to penetrate dense biofilms.	Charge-reversal nanocarriers shield cationic toxicity during mucus transit, enabling enzyme-triggered release within deep biofilms.
Aztreonam (Aztreonam lysine: Cayston)	Inhaled Antibiotic	Monobactam; binds to penicillin-binding proteins to inhibit Gram-negative bacterial cell wall synthesis.	Lyophilized powder aqueous solution degrades easily, requires immediate use post-reconstitution, and necessitates frequent nebulization (three times daily), leading to poor compliance; struggles to penetrate the mucus core.	Biodegradable liposomes protect the unstable monobactam ring, achieving sustained release to permit once-daily dosing.
Colistimethate sodium/Colistin	Inhaled Antibiotic	Cationic polypeptide; acts like a detergent to disrupt the integrity of the bacterial cell membrane.	Dry powder formulations often induce severe airway hyperresponsiveness; easily intercepted by the mucus barrier.	Cell membrane-camouflaged nanoparticles mask the irritating polypeptide, reducing airway hyperresponsiveness and improving sputum penetration.
Levofloxacin (Quinsair)	Inhaled Antibiotic	Fluoroquinolone; inhibits bacterial DNA gyrase and topoisomerase IV.	Extremely bitter taste; conventional aerosols tend to deposit in the proximal airways, failing to reach distal infection sites.	Porous nanoparticle aggregates optimize aerodynamic diameters for deep alveolar deposition while effectively masking bitter taste.
Dornase alfa (Pulmozyme)	Mucolytic Agent	Recombinant human DNase; hydrolyzes extracellular polymeric DNA released by necrotic inflammatory cells in CF sputum.	Macromolecular protein with a short in vivo half-life; exceedingly difficult to penetrate severely obstructed bronchioles when administered via conventional nebulizers.	Solid lipid nanoparticles protect fragile enzymes from proteolytic degradation, extending functional half-life and distal dispersion.
7% Hypertonic Saline	Osmotic Agent	Relies on an osmotic gradient to draw water into the airway surface liquid, hydrating mucus and promoting clearance.	Liquid aerosol droplets easily irritate the airways; cannot fundamentally degrade the macromolecular network, offering only transient action.	Hyperosmolar liposomes provide a sustained osmotic gradient, preventing acute hyperosmotic shock and severe bronchospasm.
Mannitol dry powder (Bronchitol)	Osmotic Agent	Inhaled sugar alcohol; creates a local osmotic gradient to hydrate the airways while physically stimulating cough.	Highly hygroscopic as a dry powder, prone to moisture absorption and aggregation; highly irritating to the airways, requiring a mandatory airway tolerance test prior to prescription.	Core-shell nano-assemblies utilize hydrophobic coatings to prevent premature agglomeration, improving aerosolization and mitigating acute irritation.
Pancreatic Enzyme Replacement Therapy (PERT: Creon, Zenpep, etc.)	Digestive Enzyme Replacement	Supplements exogenous lipase, protease, and amylase to ameliorate exocrine pancreatic insufficiency and fat malabsorption.	Enzyme activity is extremely sensitive to gastric acid (pH < 5.5), strictly relying on enteric-coated microsphere technology; difficult to precisely simulate physiological release rhythms.	pH-responsive nanogels withstand extreme gastric acidity and rapidly disintegrate at duodenal pH for precise enzyme release.

This table summarizes the fundamental pharmacological interventions currently approved for cystic fibrosis. Despite considerable clinical benefits, these conventional therapies exhibit significant physicochemical limitations (e.g., extreme hydrophobicity, rapid degradation, unfavorable aerodynamics) and struggle to overcome physiological barriers like the dehydrated, highly viscoelastic CF mucus and dense pathogenic biofilms. Such delivery bottlenecks highlight the crucial demand for nanoparticle-based formulations and advanced delivery vectors to optimize bioavailability, enable deep-lung penetration, and facilitate targeted intracellular or matrix-disrupting release. Abbreviations: CFTR, Cystic Fibrosis Transmembrane Conductance Regulator; CYP3A, Cytochrome P450 3 A; ΔF508, Deletion of phenylalanine at position 508

#### The pathological and biological barrier network

CF-targeted therapies rely on systemic (oral, intravenous) and pulmonary inhalation delivery [114]. Inhalation administration offers a targeted delivery route that achieves high local drug concentrations in the lungs with reduced systemic absorption, potentially improving the therapeutic ratio and minimizing off-target effects [115]. Regardless of delivery mode, CF’s aberrant microenvironment creates multi-layered biological barriers spanning anatomical, physical, chemical and cellular levels [116]. For systemic administration, pharmaceutical agents initially encounter severe pharmacokinetic and anatomical restrictions. In oral delivery, CF patients develop viscous gastrointestinal mucus, pancreatic insufficiency-driven lipid malabsorption and gut dysmotility [117, 118]. Such pathological changes reduce oral drug bioavailability [119]. Furthermore, affected individuals often exhibit altered systemic pharmacokinetic profiles. Published data report elevated volume of distribution and systemic clearance for *β*-lactams, aminoglycosides and fluoroquinolones [119]. This clinical reality mandates the administration of doses substantially higher than those used in healthy populations to maintain effective drug concentrations in the bloodstream.

A more severe challenge involves the histological barrier restricting entry from the systemic circulation into pulmonary airway infection sites [120]. The pulmonary capillary network consists of continuous endothelial cells. This dense vascular endothelial barrier strictly limits the passive extravasation of large molecular weight drugs and unmodified nanocarriers into the pulmonary interstitium [121]. After crossing endothelium into the interstitium, drugs still need to traverse compact polarized airway epithelia [122]. Drugs undergo basolateral-to-apical transcellular or paracellular transport to reach diseased airway surface liquid. CF airway lumens contain avascular mucus plugs and biofilms, leading to exponential drug concentration loss during sequential tissue penetration [123]. This severe attenuation makes it exceedingly difficult to achieve effective minimum inhibitory or therapeutic concentrations at the core of microairway lesions. Forcing an increase in systemic dosage to compensate for poor pulmonary penetrance inevitably triggers severe unintended systemic toxicities, such as nephrotoxicity and ototoxicity associated with aminoglycoside antibiotics [124].

Given the substantial limitations of systemic administration in penetrating the airway lumen, localized respiratory inhalation delivery to the CF lung presents theoretical pharmacokinetic advantages [125, 126]. It circumvents the hepatic first pass effect, greatly increases the effective drug concentration at the target site, and significantly reduces systemic side effects [127]. However, inhalation delivery similarly faces extremely harsh local biological barriers across multiple layers. The initial obstacle is a physical restriction barrier at the anatomical and aerodynamic levels. Chronic inflammation, airway remodeling and mucus plugging narrow and deform CF airway lumens [128]. These changes drastically alter the aerodynamic characteristics of inhaled aerosols. Significantly increased airflow turbulence at narrowed and complex branching sites causes larger drug particles or carriers with an aerodynamic diameter greater than five micrometers to deposit prematurely and disproportionately on the walls of the oropharynx and upper respiratory tract due to inertial impaction [129]. These particles cannot reach diseased distal bronchioles and alveoli, causing drug loss and heterogeneous pulmonary distribution [130].

The second barrier is the highly dense pathological mucus gel layer on the respiratory tract surface, which constitutes the primary microscopic challenge for local nanomedicine delivery[131]. Healthy respiratory mucus primarily consists of water, salts, and highly crosslinked mucin glycoproteins, mainly MUC5AC and MUC5B. This layer acts as a physical defense line to filter inhaled foreign matter, and its gel mesh size is typically sufficient to allow most nanoparticles to penetrate [132]. In CF, dehydrated mucus crosslinks with neutrophil-derived extracellular DNA and filamentous actin. This combination forms an extremely rigid, dense, and highly crosslinked pathological three dimensional gel network [133]. This profound biochemical alteration compresses the effective pore size of the mucus drastically to approximately 100 to 300 nm. This compression exerts a strong steric hindrance effect on any drug loaded particles physically larger than this effective pore size, directly intercepting them at the surface of the mucus mesh. Beyond steric restriction, mucus imposes additional interactive retention effects. Because highly glycosylated mucins and the phosphate backbone rich extracellular DNA carry a strong net negative charge at physiological pH, they rapidly lock onto and physically capture any positively charged drug molecules or cationic nanocarriers through electrostatic attraction [134]. Concurrently, the widely distributed hydrophobic domains on mucin fibers firmly adhere poorly hydrophilic drug particles through strong hydrophobic interactions and multiple hydrogen bond entanglements. Immobilized drugs are eliminated via residual muco-ciliary clearance or cough[134]. This causes the therapeutic agents to completely lose their potential to cross the mucus layer and contact the underlying target airway epithelial cells [135].

The third exceptionally insurmountable obstacle is the refractory microbial biofilm barrier constructed by pathogenic bacteria within the hypoxic airways. Chronic lower-airway colonization by *Pseudomonas aeruginosa* triggers quorum-sensing-mediated gene shifts and phenotypic switching [136]. They begin to synthesize and secrete massive amounts of extracellular polymeric substances primarily composed of high molecular weight alginate. This tough and highly viscoelastic three dimensional protective shield built by the bacteria themselves firmly encases massive bacterial microcolonies on the mucosal surface, effectively resisting phagocytosis by host macrophages and neutrophils [137]. It also forms a physicochemical barrier that is extremely difficult to penetrate, greatly hindering the diffusion of conventional antibiotics and unmodified nanocarriers deep into the biofilm [138]. More fatally, a highly heterogeneous microenvironment exists inside the biofilm, containing steep gradients of oxygen, nutrients, and pH. Nutrient and oxygen deprivation drives central biofilm bacteria into metabolically dormant persister phenotypes [139]. Because the vast majority of bactericidal antibiotics commonly used clinically target actively dividing bacteria by interfering with cell wall synthesis or nucleic acid replication, these dormant persister cells can survive under the sustained attack of extremely high antibiotic concentrations [140]. This makes the overall tolerance of bacteria within CF biofilms to conventional antimicrobial drugs hundreds to a thousand times higher than in their free floating planktonic state, serving as the fundamental microbiological root cause for the periodic outbreaks of deep pulmonary infections and medical treatment failures.

Finally, for the few therapeutic particles that penetrate the dual physical interception of the highly viscous mucus layer and dense biofilm through precise physicochemical design, they still face a rigorous cytological clearance barrier constructed by the lower respiratory immune system before reaching target cells [141, 142]. Alveolar macrophages act as the frontline of pulmonary innate immune defense, accounting for more than 90% of the resident immune cells in the respiratory tract [143]. They possess extremely high migratory and surveillance activity across the vast alveolar surface, capable of rapidly recognizing, engulfing, and utilizing lysosomal enzymes to degrade large sized inhaled microparticles through efficient phagocytosis. In addition, alveolar surfactant lipids and proteins adsorb onto particles to form a protein corona [144]. This structure acts as an opsonin to further accelerate the specific recognition and destruction of drug loaded systems by macrophages, thereby drastically shortening the half-life of drugs in the lungs. For non-viral nanocarriers aimed at introducing functional nucleic acids into airway epithelial cells to fundamentally correct gene defects, they must also overcome a series of stringent subcellular level barriers [145]. These carriers resist extracellular nuclease degradation, enter epithelia via endocytosis, and escape endosomal degradation before cargo breakdown. Ultimately, they must transport the extremely fragile genetic payload intact into the cytoplasm or nucleus to achieve stable gene expression [146].

In summary, whether addressing the systemic pharmacokinetic alterations and cross vascular epithelial basolateral transport obstacles faced by systemic administration, or the severe aerodynamic changes, highly viscous mucus networks, highly resistant bacterial biofilms, and rigorous macrophage immune surveillance systems encountered by local inhalation administration, these deeply intertwined multidimensional and multilevel biological defense lines collectively constitute the core challenge for current systemic and pulmonary targeted drug delivery and nanomedicine translation in CF.

### CFTR modulators

Over the past several decades, the therapeutic landscape of CF has experienced a transformative evolution following the discovery and clinical integration of CFTR modulator APIs. These specific APIs constitute the absolute core of disease modifying therapies for CF. Deviating from conventional symptomatic management, disease modifying therapy agents intervene directly upstream in the disease pathogenesis. They precisely target the fundamental defects in channel protein folding, trafficking, gating, or synthesis that arise from CFTR genetic mutations. At present, they represent the exclusive pharmacological class capable of fundamentally altering, delaying, or even reversing the natural progression of the disease.

Guided by their distinct mechanisms of action at the cellular and molecular levels, this API category is systematically subdivided into five core classifications: (1) CFTR potentiators, (2) CFTR correctors, (3) CFTR readthrough agents, (4) CFTR stabilizers or amplifiers, and (5) CFTR splice modulators or antisense oligonucleotides [105]. Despite demonstrating remarkable efficacy during clinical trials, the conventional oral formulations of these CFTR modulators, such as tablets or granules, suffer from intractable pharmacokinetic and pharmacodynamic limitations [73]. Consequently, the ensuing section will meticulously examine each subclass to elucidate how advanced nanotechnology can optimize their delivery capabilities and shape their future developmental trajectories.

#### CFTR potentiators

CFTR potentiators, primarily exemplified by ivacaftor, represent the foundational APIs in CF disease-modifying therapies [147]. This agent acts as a pharmacological chaperone to allosterically stabilize the open conformation of defective channels at the apical membrane [148, 149]. However, its extreme lipophilicity dictates profound pharmacokinetic constraints during conventional oral administration [150, 151].

**Fig. 5:**
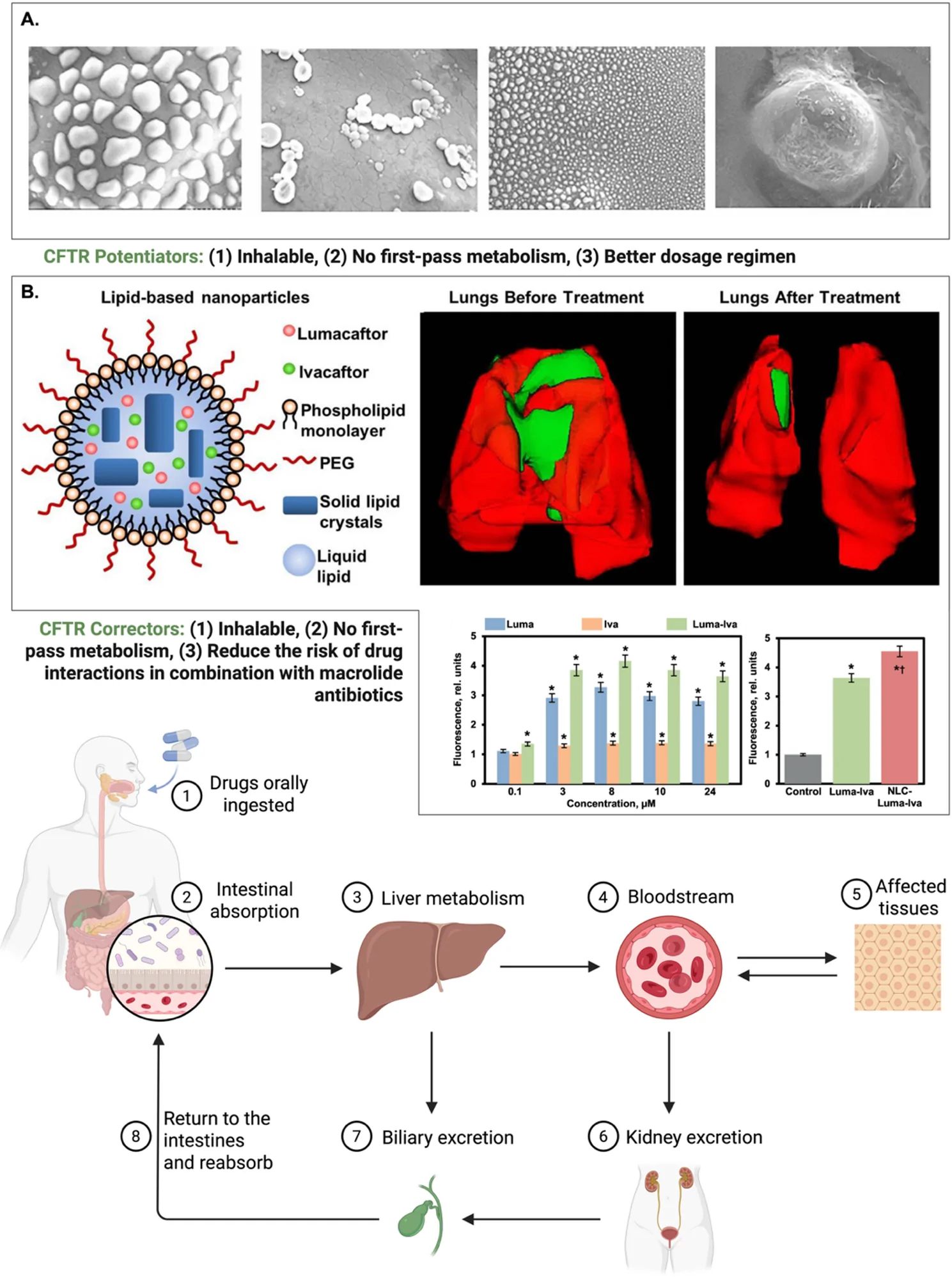
Therapeutic Platform of Inhalable Nanoparticle-encapsulated Cystic Fibrosis Transmembrane Conductance Regulator (CFTR) for Cystic Fibrosis (CF) Treatment. **(A)** Scanning electron microscopy images showing the morphological characteristics of CFTR modulator-loaded nanoparticles optimized for pulmonary delivery [152]. **(B)** Schematic of lumacaftor/ivacaftor co-loaded PEGylated nanostructured lipid carriers, in vivo lung imaging of CF mice pre- and post-treatment, oral drug disposition pathway, and in vitro assay results verifying CFTR functional restoration [156]. Reproduced with permission

Inhalable nanocarrier delivery architectures offer a revolutionary optimization pathway to overcome this translational bottleneck of excessive systemic distribution paired with inadequate local organ concentrations. Recent pharmaceutical developments have yielded surface-engineered ivacaftor nanoparticles constructed from a polylactic acid and chitosan matrix (Fig. 5A ) [152]. Meticulous formulation adjustments strictly confine the geometric diameter of these drug-loaded nanocarriers to under 300 nm, complemented by surface PEGylation. This specific surface engineering grants the carriers excellent hydrophilic stealth characteristics. Such properties effectively diminish nonspecific binding interactions with the dense, highly concentrated mucin networks found in CF airways, empowering the particles to efficiently breach the mucus barrier and reach the underlying epithelial targets. Aerodynamic evaluations confirm that the mass median aerodynamic diameter of the lyophilized powder stabilizes between 4.75 and 5.39 μm. These dimensions perfectly satisfy the aerodynamic criteria necessary for achieving high fine particle fractions and ensuring robust deposition within the deep alveolar and bronchiolar regions. Carrying profound clinical implications, the polymeric matrix establishes zero-order sustained-release kinetics that continuously deploy ivacaftor over a 24 to 36-h window. This localized nano-therapeutic intervention not only presents a viable route to condense standard multi-dose regimens into a single daily inhalation but also maximizes precision airway therapy while completely circumventing the hepatic metabolic burden.

#### CFTR Correctors

CFTR correctors serve as the core disease-modifying agents developed against the most prevalent class II mutations in CF, classically represented by the ΔF508 variant. Representative API in this category include first-generation lumacaftor and tezacaftor, alongside the next-generation elexacaftor [153]. At the molecular pathology level, the ΔF508 mutation induces severe thermodynamic instability and misfolding of the nascent CFTR protein within the endoplasmic reticulum. Intracellular molecular quality control systems rapidly recognize this aberrant conformation [154]. Subsequently, the protein undergoes ubiquitination and is directed toward the proteasomal pathway for complete degradation. This cascade results in a profound depletion of functional ion channels at the apical membrane of epithelial cells. Functioning as pharmacological chaperones, CFTR correctors bind specifically to the transmembrane domains of the mutated CFTR protein to thermodynamically stabilize its native conformation. This vital interaction assists the mutant proteins in evading endoplasmic reticulum degradation checkpoints, facilitating their successful trafficking to the Golgi apparatus for glycosylation and ultimate anchorage at the epithelial cell surface. Because the rescued proteins typically retain inherent gating defects, clinical protocols mandate a combinatorial intervention strategy utilizing both correctors and potentiators. Revolutionary regimens like the triple-combination therapy Trikafta exemplify this approach. They achieve a synergistic multiplier effect that integrates structural rescue at the endoplasmic reticulum with robust channel activation and amplification at the membrane surface.

During clinical translation, these high-molecular-weight APIs with their complex polycyclic structures exhibit extreme lipophilicity and exceptionally poor aqueous solubility [155]. Their conventional oral formulations encounter severe bioavailability bottlenecks, characterized by highly erratic absorption and a strict dependence on high-fat diets to facilitate systemic uptake. More critically, massive systemic exposure triggers intense hepatic first-pass metabolism mediated by the cytochrome P450 enzyme system, specifically CYP3A4 and CYP3A5. This metabolic degradation severely restricts the effective drug concentration reaching the pulmonary target organs. It simultaneously introduces substantial risks of hepatobiliary toxicity and highly complex drug-drug interactions.

To definitively overcome these physicochemical and pharmacokinetic barriers, nano-delivery systems targeting the respiratory mucosa have emerged as a highly promising solution. Recent breakthrough investigations have utilized nanostructured lipid carriers characterized by an imperfect crystal lattice to successfully achieve the highly efficient pulmonary co-delivery of lumacaftor and ivacaftor (Fig. 5B) [156]. Compared to first-generation solid lipid nanoparticles (LNPs) that frequently expel loaded drugs during recrystallization phases, nanostructured lipid carriers are constructed from a precise mixture of solid and liquid lipids. This compositional strategy generates a high degree of microscopic physical disorder within their internal matrices. Such structural disorder provides immense hydrophobic encapsulation space for the extremely lipophilic CFTR modulator components, thereby effectively preventing drug recrystallization and leakage during prolonged storage. Following surface chemical engineering modifications designed to enhance mucus penetration, these lipid nanocarriers can seamlessly traverse the highly concentrated pathological sputum present in CF airways. Direct nebulized inhalation allows them to establish an exceptionally high localized concentration gradient directly at the apical membrane of the airway epithelium. The integration of this cutting-edge nanotechnology decreases the absolute administered dose required for equivalent targeted rescue by several orders of magnitude. It also fundamentally bypasses hepatic first-pass metabolic conflicts and systemic hemodynamic distribution. Ultimately, this innovation offers a next-generation disease-modifying therapy delivery model that combines paramount biosafety with precise organ targeting. It proves particularly beneficial for CF patients who cannot tolerate oral formulations due to severe hepatic impairment or those who require prolonged co-administration of macrolide antibiotics to manage complex infections.

#### Other CFTR modulators

CFTR readthrough agents, stabilizers or amplifiers, and splicing modulators constitute the next generation of disease modifying therapies extending beyond contemporary small molecule modulators [157]. Nevertheless, their clinical translation is universally hindered by suboptimal delivery efficiencies. Although the nanocarrier strategies for these three pharmacological classes remain at disparate developmental stages, they collectively converge upon inhalable nano-delivery as the central translational pathway.

Readthrough agents, exemplified by ataluren and ELX 02, confront the profound translational hurdles of exceptionally low oral bioavailability and inadequate pulmonary exposure. Recent investigations demonstrate that LNPs have achieved significant breakthroughs in delivering gene editors to repair nonsense mutations [158]. Specifically, Tafech and colleagues utilized optimized LNPs to deliver CRISPR systems into patient derived cells harboring the R1162X mutation, achieving a genetic correction rate of approximately 12%. This milestone provides critical conceptual insights for the nano-delivery of readthrough agents. While research investigating chitosan nanoparticles directly encapsulating ataluren remains in the preliminary exploratory phases, the LNP platform exhibits definitive potential for managing nucleic acid based readthrough molecules, such as tRNA derivatives [105].

The exploration of delivery systems for stabilizers and amplifiers has advanced toward an innovative technical framework. When administered in conjunction with clinical correctors, this approach synergistically elevates protein maturation and channel functionality. Such a strategy perfectly actualizes the amplifier concept, facilitating sustained functional maintenance through the continuous intracellular expression of mRNA encoded protein therapeutics. Furthermore, it establishes a foundational platform for the delivery of alternative stabilizing molecules, including PDE5 inhibitors [5]. LNPs successfully mediate highly efficient intracellular delivery post inhalation, while the nanobodies represent an entirely novel molecular modality for stabilizing agents.

As antisense oligonucleotide (ASO) therapeutics, splicing modulators exhibit the most stringent dependence on nanocarrier technologies. SpliSense’s SPL84 has emerged at the forefront of this developmental landscape [20]. Preclinical evaluations confirm that this inhalable ASO can effectively penetrate the highly viscous sputum layers characteristic of CF patients and undergo subsequent cellular uptake and nuclear translocation within the airway epithelium. Phase I clinical trials have further corroborated its excellent safety and tolerability profiles. Addressing the W1282X nonsense mutation, which concurrently presents with splicing aberrations, Kim and colleagues implemented an exon skipping ASO strategy to restore CFTR function at the cellular level. However, because these ASOs must access the nucleus to interact with precursor mRNA, they impose substantially elevated requirements for endosomal escape efficiency upon the delivery vectors.

In summary, the nanotechnological trajectories for these three therapeutic classes exhibit a differentiated yet mutually illuminating landscape. Readthrough agents anticipate the strategic extension of LNP platforms from gene editing applications toward small molecule and nucleic acid readthrough molecules. Stabilizers and amplifiers are currently leveraging mRNA LNP technologies to pioneer a novel strategy for pulmonary protein expression. Meanwhile, splicing modulators rely on SPL84 as a definitive benchmark to validate the clinical feasibility of inhalable ASOs. The overarching consensus dictates that nanocarriers are evolving from rudimentary delivery vehicles into highly sophisticated functional delivery systems. Beyond merely shielding pharmacological agents across the mucosal barrier and facilitating endocytosis and endosomal escape, these advanced platforms provide comprehensive solutions for combined delivery, spatiotemporal controlled release, and sustained expression. Ultimately, these structural innovations will propel these root cause interventions from theoretical concepts into definitive clinical realities.

**Fig. 6:**
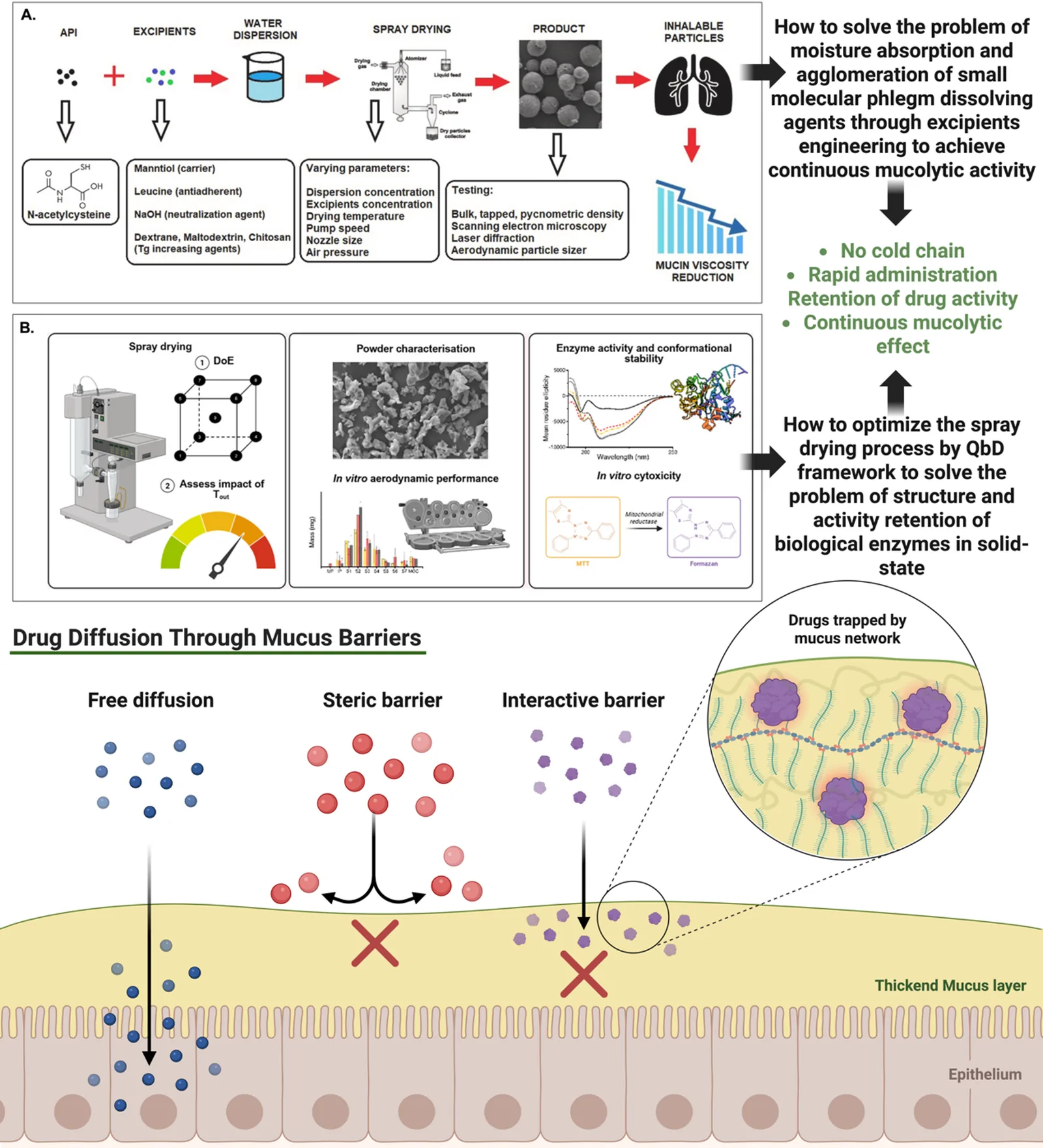
This Schematic Illustrates the Rational Development, Process Optimization, and Underlying Pulmonary Delivery Barriers of Inhalable Dry Powder Mucolytic Formulations for Cystic Fibrosis (CF) Therapy. **(A)** Excipient engineering strategy and spray drying manufacturing workflow of inhalable N-acetylcysteine microparticles, which addresses moisture absorption and particle agglomeration of small-molecule mucolytics to achieve sustained mucolytic activity [159]. **(B)** Quality-by-design (QbD)-guided spray drying process optimization of deoxyribonuclease I dry powder for inhalation, focusing on the structural integrity and enzymatic activity retention of the biologic enzyme in the solid state [160]. Reproduced with permission

### Mucolytics and airway clearance agents

Dehydrated mucus within the CF airway arises from abnormal ion transport and contains high concentrations of free DNA alongside cross-linked mucins [161]. This pathological matrix constitutes the ultimate physical barrier impeding the deep penetration of all inhalation therapies. Although recombinant human deoxyribonuclease I (rhDNase I) and N-acetylcysteine (NAC) serve as the primary pharmacological agents to disrupt this barrier, conventional liquid nebulized formulations suffer from severe clinical translation bottlenecks [162, 163]. These limitations include strict cold chain requirements, long administration times, and rapid muco-ciliary clearance from the respiratory tract. Thus, dry powder inhalation (DPI) systems and advanced particle engineering have emerged as essential solutions to address this pharmaceutical challenge.

Translating macromolecular biological enzymes into a solid state presents a fundamental challenge regarding the preservation of their three-dimensional protein structures during the extreme physical stresses of spray drying. Investigators have systematically optimized the co-spray drying process for deoxyribonuclease I by implementing a Quality by Design (QbD) framework (Fig. 6B) [160]. This engineering reconfiguration endows the resulting powder with exceptional aerodynamic properties to facilitate deep pulmonary deposition. Furthermore, it completely eliminates the necessity for cold chain logistics while remarkably preserving up to 94% of the inherent enzymatic degradation activity [164]. Such advancements establish a new benchmark for the highly efficient, portable, and noninvasive delivery of exceedingly fragile macromolecular muco-regulators.

Small-molecule mucin depolymerizing agents frequently exhibit detrimental physicochemical tendencies toward hygroscopicity and agglomeration during the micronization process. To address these defects, precision excipient engineering demonstrates immense translational value. During the development of NAC dry powders, researchers successfully engineered inhalable microparticles possessing an optimal morphology and a mass median aerodynamic diameter of approximately 5.95 μm (Fig. 6A) [159]. This was achieved by incorporating 20% to 25% leucine to function as an anti-adherent alongside mannitol to regulate osmotic pressure. Crucially, this microparticulate strategy improves the pulmonary reach of the therapeutic agent while achieving sustained mucolytic activity for up to 120 min in vitro. This extended action promoted a profound 35.3% reduction in mucus viscosity. This excipient-driven mechanism of sustained viscosity reduction definitively compensates for the primary vulnerability of conventional liquid formulations, which are notoriously susceptible to rapid muco-ciliary clearance [165].

Looking ahead, therapeutic strategies for airway clearance are undergoing a fundamental conceptual shift. The focus is shifting from the passive degradation of preexisting mucus networks toward the active inhibition of their formation at the source. Emerging therapeutic strategies will rely on advanced nanocarriers to navigate formidable pathological barriers and precisely deliver nucleic acid therapeutics targeting the expression of MUC5AC or MUC5B. This intervention aims to completely halt the hypersecretion of pathogenic mucins at the transcriptional level. This will advance the field of muco-regulatory agents beyond symptomatic relief to true disease modification.

### Nucleic acids and gene therapies

Nucleic acid and gene-based therapeutics stand at the forefront of modern precision medicine, providing a powerful mechanism to target and resolve genetic respiratory diseases directly at their source [166]. While gene therapy offers the profound potential to correct the genetic etiology of CF or remodel the local inflammatory microenvironment, clinical translation has been continuously impeded by the absence of optimal in vivo delivery vectors [167]. This promising approach is fundamentally limited by a critical physiological barrier. Therapeutic cargoes, encompassing both gene replacement constructs and gene-editing machineries, must successfully breach the pathologically hyper-viscous mucosal barrier lining the CF airways to transfect the target pulmonary epithelium [168]. Developing safe and highly efficient non-viral nanocarrier systems capable of withstanding this complex pathological milieu is thus imperative [169]. Overcoming the structural and cellular barriers of the deep lung remains the definitive prerequisite for unlocking the true clinical efficacy of nucleic acid and gene therapies in CF populations.

#### Gene expression and replacement

Gene replacement therapy aims to introduce a functional copy of the CFTR gene into target respiratory cells, fundamentally restoring ion transport function at its source [170]. As therapeutic cargo, mRNA offers distinct advantages over conventional plasmid DNA (pDNA) [171, 172]. It bypasses the nuclear membrane barrier and eliminates the risk of genomic integration, resulting in rapid expression and an enhanced safety profile. However, the extreme susceptibility to degradation and the transient expression characteristics inherent to mRNA impose stringent requirements on nanocarriers. These vectors must provide robust in situ protection and ensure safety during repeated administrations. This dynamic interplay between the formulation and the physiological microenvironment has driven the evolution of multidimensional nano-delivery strategies.

**Fig. 7:**
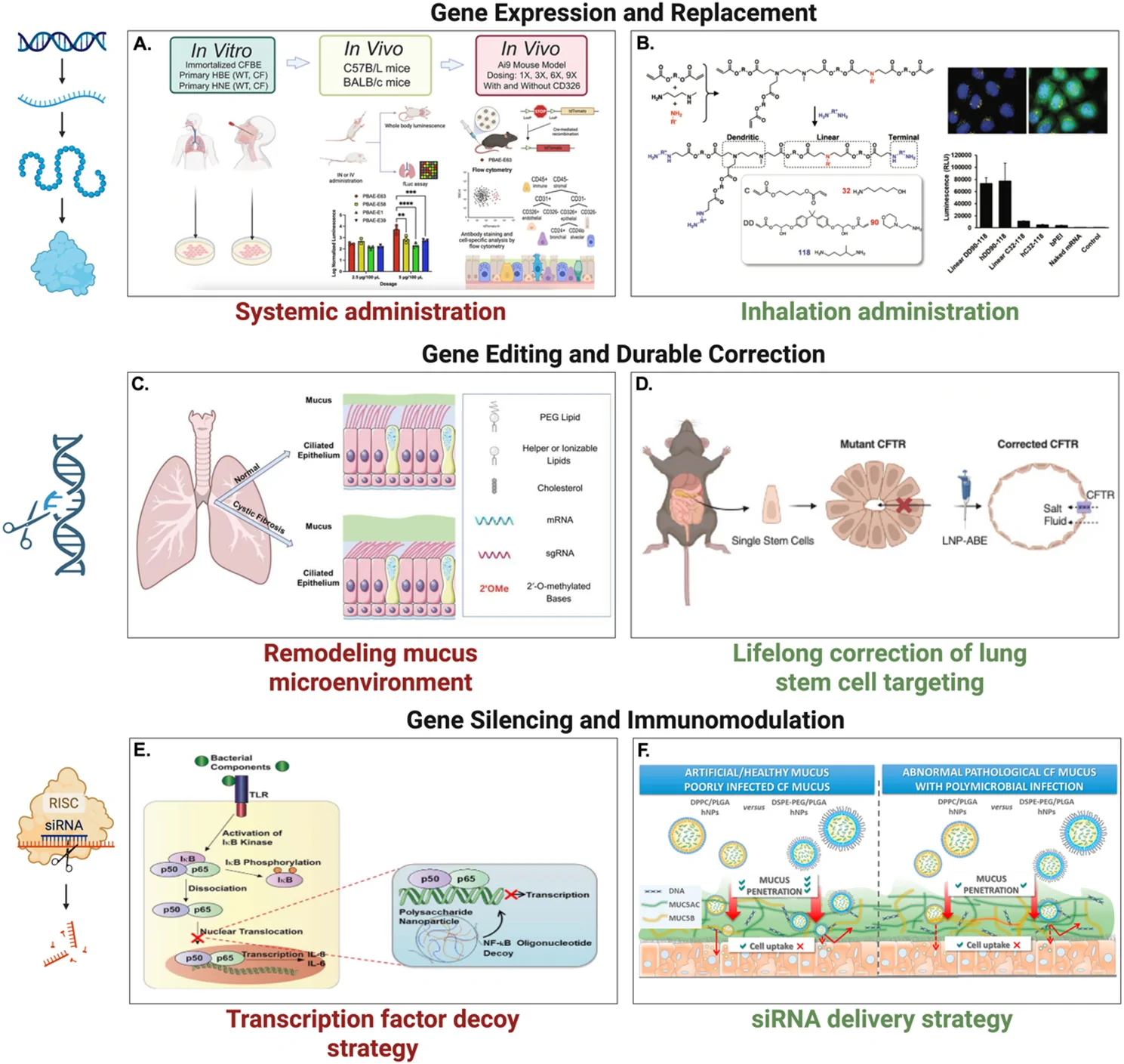
Schematic Overview of Non-viral Nanocarrier-mediated Gene Therapeutic Strategies for Cystic Fibrosis (CF) Treatment. This figure summarizes three core therapeutic approaches for CF, including gene expression and replacement, gene editing and durable correction, and gene silencing and immunomodulation. **(A)** In vitro and in vivo evaluation workflows for systemic administration of nanocarriers for CF transmembrane conductance regulator (CFTR) gene replacement therapy [174]. **(B)** Chemical optimization, transfection efficiency validation, and functional assessment of nanocarriers via inhalation administration for airway-localized gene delivery [173]. **(C)** Nanocarrier engineering strategies to remodel the CF mucus microenvironment for enhanced gene editing delivery across the mucosal barrier [179]. **(D)** Lung stem cell-targeted lipid nanoparticle (LNP) gene editing systems to achieve lifelong correction of pathogenic CFTR mutations, with organoid and in vivo efficacy validation [180]. **(E)** Transcription factor decoy oligonucleotide delivery strategy for immunomodulation of CF-related airway inflammation [183]. **(F)** Small interfering RNA (siRNA) delivery platform for targeted gene silencing to mitigate hyperactive inflammatory signaling in CF airway [184]. Reproduced with permission

For local nebulized inhalation, nano-formulated hyperbranched poly(beta-amino ester)s (hPBAEs) have demonstrated exceptional potential to withstand aerosolization shear forces and penetrate the respiratory mucus barrier (Fig. 7A) [173]. In vitro and in vivo evaluations indicate that inhaled hPBAE-mRNA complexes achieve uniform distribution across all five murine lung lobes. Twenty-four hours after a single dose, pulmonary target protein expression reached 101.2 ng/g, yielding a successful transfection rate of approximately 24.6% within the total lung epithelial cell population. Importantly, this formulation supports the repeated dosing required for sustained protein expression without causing detectable local or systemic toxicity.

However, topical inhalation faces physical limitations when encountering the severely thickened and frequently obstructed pathological airways characteristic of end-stage CF patients, preventing the therapy from reaching deep-seated lesions. Systemic intravenous administration provides a strategic alternative to circumvent this anatomical roadblock. By optimizing end-modified biodegradable poly(beta-amino ester) (PBAE) nanoparticles, Erin W Kavanagh et al. have confirmed that these carriers can efficiently cross the vascular endothelial barrier post-injection (Fig. 7A) [174]. This enables robust targeted mRNA delivery and subsequent expression within the pulmonary bronchial, epithelial, and endothelial cell populations, all without relying on complex surface targeting ligands.

Additionally, to address the intrinsic discrepancy in overall transfection efficiency between non-viral and viral vectors, novel peptide-poloxamine self-assembling nanoparticles have emerged as a highly versatile delivery platform [175]. Through modular peptide design, this system significantly amplifies mRNA expression both in vitro and in vivo. It also safely mediates the delivery of pDNA, resulting in the enduring and effective repair of CFTR defects in the lungs of CF mice with negligible toxicity. The adaptability inherent in this formulation design demonstrates that iterative advancements in polymeric nanotechnology are systematically dismantling the dual barriers of poor targeting and short half-lives that have historically hindered nucleic acid replacement therapies.

#### Gene editing and durable correction

Gene replacement therapies confer merely transient functional protein expression. Conversely, genome editing endeavors to achieve a permanent, sequence-level rewriting of pathogenic mutations [176]. Within this highly challenging clinical translation, nanotechnology assumes an indispensable central role. It serves as the foundational vehicle necessary to overcome the in vivo delivery barriers associated with the bulky CRISPR system [177].

During the fundamental design phase of nanoformulations, the physicochemical state of the therapeutic cargo exerts a decisive impact on the ultimate efficiency of genetic repair. CF pathological cells are characterized by diminished metabolic rates. Encapsulating the Cas9 editing tool as a ribonucleoprotein complex within LNPs, rather than relying on bare mRNA, effectively bypasses the inefficient translational bottlenecks inherent to these target cells [178]. In vitro data robustly substantiate this approach, with nanoparticles loaded with Cas9 protein achieving a homology-directed repair rate of up to 4.8% in ΔF508 mutant cells. Under equivalent dosing conditions, mRNA nanoformulations resulted in a homology-directed repair rate of nearly zero. Such precise optimization of cargo loading based on nanocarriers establishes the pharmacological foundation for achieving effective protein functional restoration.

However, transitioning the perspective from the molecular level to complex pathological tissues reveals severe physiological impediments associated with local nebulized administration. According to the design of Belal Tafech et al., biomimetic 3D human CF bronchial tissue models exhibit an abnormally dense and viscous pathological mucus barrier (Fig. 7C) [179]. Constrained by this barrier, the editing efficiency of conventional topically applied LNPs experiences a precipitous decline from 50% in 2D monolayers to approximately 5%. Confronted with this physical bottleneck, relying solely on iterative improvements to the nanomaterials frequently yields marginal benefits. A strategic approach shift involves remodeling the targeted microenvironment. Pretreatment with the clinically approved mucolytic agent dornase alfa substantially improves the tissue penetrance of nanoparticles, thereby doubling the gene editing efficiency to 12.7% against the prevailing odds. Leveraging this combinatorial nano-delivery strategy, they successfully achieved approximately 12% precise base editing correction of the refractory R1162X nonsense mutation in patient-derived cellular models.

Despite notable advancements in breaching local delivery barriers, the inherent continuous desquamation and renewal of the respiratory epithelium dictate that repairing superficial mature cells confers only temporary clinical benefits. Achieving genuine “Durable Correction” mandates that therapeutic interventions precisely access and rewrite the lung stem cells situated at the basal layer of the airways. In this context, systemically administered, organ-selective nanoparticles demonstrate exceptional potential to disrupt conventional limitations. A landmark study provides the first in vivo confirmation of this capability (Fig. 7D) [180]. Highly optimized lung-targeting LNPs efficiently cross the vascular endothelial barrier to achieve over 70% genomic editing in murine lung stem cells. Furthermore, the healthy epithelial cell populations differentiated from these edited stem cells remain stable for up to 660 days. Operating within an extremely rigorous in vivo environment, these advanced nanocarriers successfully mediated the correction of the R553X mutation in 50% of the lung stem cells in CF mice. This intervention substantively restored the ion channel function of the CFTR protein. This in vivo stem cell targeting strategy, driven by nanotechnology, fundamentally clears the ultimate translational pathway for gene therapies to progress toward single-dose administration and lifelong curative outcomes.

#### Gene silencing and immunomodulation

In the pathological progression of chronic respiratory diseases such as CF, merely correcting the underlying genetic defect often fails to immediately reverse established hyperinflammation and mucus obstruction. Utilizing nucleic acid technologies to mediate gene silencing and immunomodulation has increasingly become a critical intervention for remodeling the airway microenvironment [181]. Throughout this highly challenging translational process, nanotechnology serves not only as a physical vehicle to overcome epithelial uptake barriers but also acts as the core driver determining the success of targeted therapies [182].

To address complex inflammatory cascades, investigators have expanded beyond the traditional scope of RNA interference by innovatively engineering polysaccharide nanoparticles (PSA-TMC) coated with NF-κB decoy oligonucleotides (Fig. 7E) [183]. Within bronchial epithelial inflammation models induced by pathogen-derived lipopolysaccharides, this nanoformulation competitively blocks transcription factor activation. This mechanism significantly downregulates the secretion of key proinflammatory mediators, including IL-6 and IL-8. Such nanostrategies offer precise pharmacological validation for directly intervening in exaggerated airway immune responses.

When attempting to directly silence inflammatory pathways using small interfering RNA (siRNA), the surface chemical modification of nanoparticles requires rigorous dialectical evaluation. Traditional pharmaceutics generally considers PEGylation the gold standard for enhancing mucus penetration. However, a recent study utilizing hybrid lipid-polymer nanoparticles (hNPs) has uncovered starkly different dynamic behaviors within authentic sputum samples from CF patients. Inside exceptionally dense and polymicrobial-colonized pathological mucus, non-PEGylated hNPs demonstrated a barrier-penetrating capacity far superior to their PEGylated counterparts. They subsequently achieved highly efficient NF-κB gene knockdown in lipopolysaccharide-stimulated epithelial cell models (Fig. 7F) [184]. This counterintuitive discovery profoundly illustrates that the physicochemical design of nanocarriers must avoid dogmatic assumptions and instead perfectly match the specific pathological microenvironment.

Translating cellular-level gene silencing potential into tangible clinical benefits also requires overcoming the engineering hurdles of physicochemical instability inherent to liquid formulations. Recent pharmaceutical advancements have successfully navigated this challenge through spray drying techniques. Protected by a 5% lactose excipient solution, siRNA LNPs were effectively converted into dry powder inhalers that possess excellent aerodynamic properties. Upon redispersion, these nanoparticles reliably maintained an optimal diameter of approximately 150 nm while entirely preserving the biological activity of their internal nucleic acids. Evaluated in the highly translatable ex vivo human precision-cut lung slice (PCLS) model, this dry powder formulation sustained the robust protein downregulation of over 90% seen in standard in vitro models. It further achieved up to 50% target gene silencing without provoking any aberrant cytokine release [185]. This nanotechnology-driven formulation innovation definitively dismantles the translational barriers preventing the noninvasive administration of nucleic acid immunomodulators deep into the lower respiratory tract.

The preceding chapters have systematically delineated the molecular mechanisms and nanocarrier engineering advances of three major categories of nucleic acid therapeutics: gene expression replacement, gene editing, and gene silencing and immunomodulation, validating the breakthrough potential of non-viral nanocarriers in overcoming the CF airway mucus barrier and achieving pulmonary tissue-targeted delivery. However, existing discussions have predominantly focused on performance optimization of individual technologies and have failed to clearly delineate the clinical indication boundaries, therapeutic durability differences, delivery endpoint requirements, and risk-benefit profiles of these major strategies: mRNA, pDNA, siRNA, and gene editing. Critical analysis reveals that the advantages and limitations of these strategies exhibit highly asymmetric characteristics: while mRNA replacement therapy possesses the core strengths of no genomic integration risk and broad applicability across all genotypes, its 2–4 week duration of protein expression following a single dose is frequently underestimated in existing studies, and the economic burden and patient adherence issues associated with lifelong monthly repeated administration constitute an unavoidable core barrier to its large-scale clinical application. Although CRISPR gene editing is widely claimed to have single-dose curative potential, this conclusion is based solely on short-term data from murine models; its long-term carcinogenic risk from off-target editing and clonal expansion risk following lung stem cell editing remain inadequately validated. Furthermore, it is only applicable to early-stage pediatric patients with no irreversible airway structural damage and offers negligible therapeutic value to the vast majority of adult patients who constitute the bulk of the clinical population. As adjuvant therapeutic modalities, siRNA exerts only anti-inflammatory and muco-regulatory effects that alleviate symptoms but cannot reverse disease progression, and their cumulative renal and hematological toxicity from long-term repeated administration has not been fully evaluated in long-term clinical trials. More critically, existing studies have universally overemphasized optimization of nanocarrier delivery efficiency yet have deliberately overlooked the core clinical reality that complete airway obstruction in end-stage CF patients renders inhaled administration entirely ineffective. This research orientation, which is disconnected from real-world clinical scenarios, is leading to substantial misallocation of translational resources.

#### Nanocarrier engineering for nucleic acid delivery

In the clinical translation of nucleic acid therapeutics, nanotechnology no longer functions merely as a passive physical vehicle. Instead, its fundamental materials engineering design has emerged as the core determinant of delivery success[186]. Departing from prior discussions that prioritized in vivo biological expression outcomes, the dialectical focus of this section centers on how nanoscale chemical and physical structural modifications actively adapt to and even remodel formidable physiological barriers [187].

**Fig. 8:**
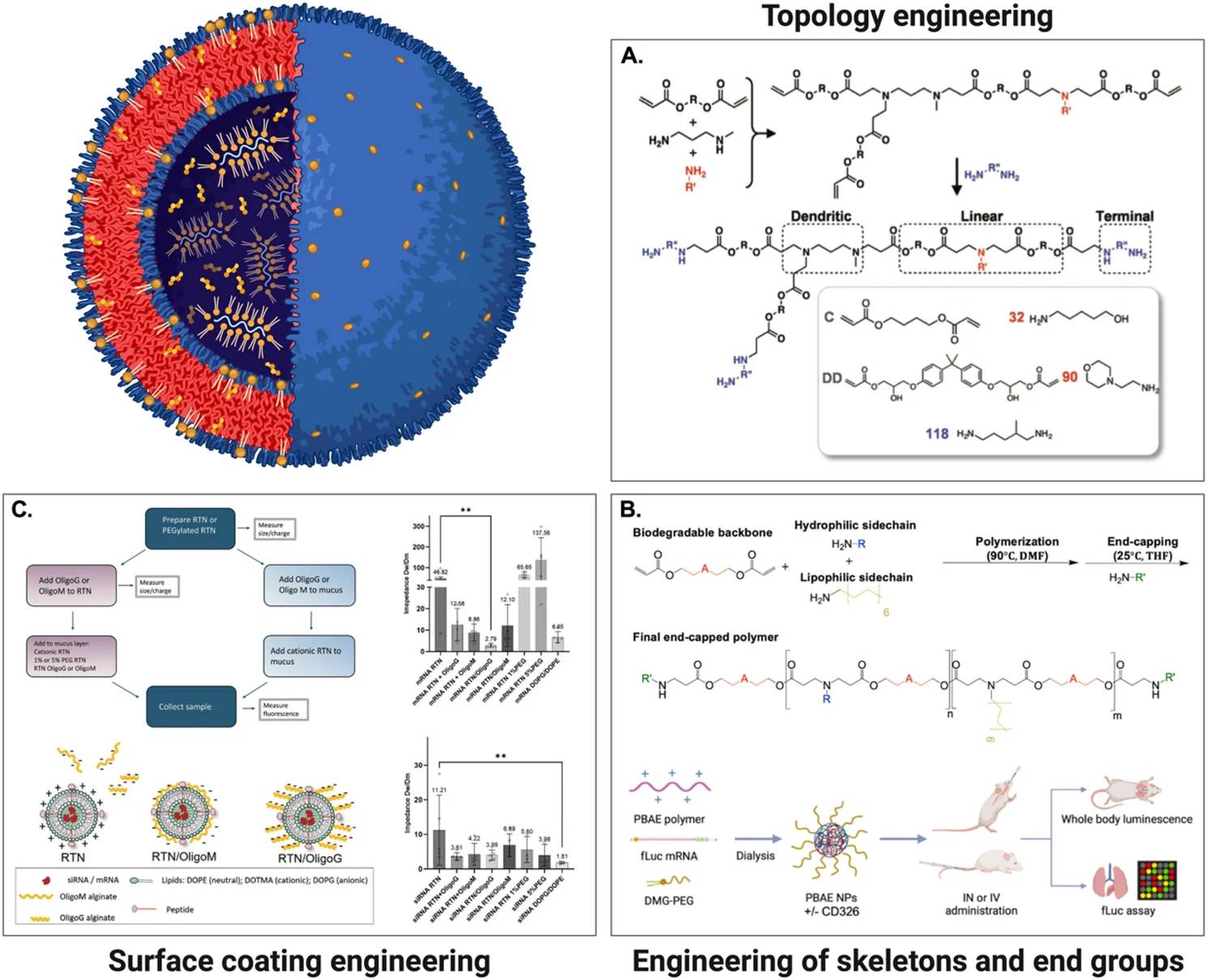
Schematic Overview of Structure-guided Engineering of Non-viral Nanocarriers for Efficient Nucleic Acid Delivery to The Lungs. **(A)** Topology engineering of hyperbranched poly(β-amino ester) (PBAE) polymers for stable inhalable mRNA polyplexes [173]. **(B)** Backbone and end-cap group engineering of PBAE nanoparticles to achieve ligand-free lung-targeted systemic mRNA delivery [174]. **(C)** Surface coating engineering of receptor-targeted nanocomplexes with alginate oligosaccharides to overcome the cystic fibrosis mucus barrier [188]. Reproduced with permission

First, conventional linear polymer encapsulation proves inadequate for overcoming the extreme physical shear forces generated during nebulized administration. Second, traditional strategies regarding organ targeting via systemic intravenous administration rely heavily on complex surface receptor-ligand conjugations. Here, we outline how two previously highlighted investigations resolve these dual challenges. The former study utilized precise topological engineering to innovatively synthesize hPBAEs (Fig. 8A) [173]. This three-dimensional polymeric network endows mRNA nanocomplexes with exceptional spatial compactness and mechanical stability. Consequently, this materials physics approach fundamentally prevents structural dissociation and premature nucleic acid leakage during aerosolization. The latter research applied precise chemical end-group modifications to biodegradable PBAEs (Fig. 8B) [174]. Researchers demonstrated that the structural evolution of the basic carrier backbone alone can achieve highly efficient targeted accumulation within pulmonary bronchial and epithelial cell populations without requiring any exogenous targeting ligands. This ligand-free architecture significantly streamlines the pharmaceutical development trajectory of these nanocarriers.

Finally, within the realm of surface coating engineering designed to traverse dense pathological mucus, classical PEGylation strategies frequently face a compromising trade-off that sacrifices ultimate cellular uptake and transfection efficiency. By introducing low molecular weight alginate oligosaccharides, specifically OligoM, to remodel the surface of cationic nanocomplexes, this novel non-PEGylated engineered coating demonstrates remarkable superiority. It not only achieves highly efficient diffusional penetrance through static CF mucus barriers but also mediates mRNA transfection efficiencies that significantly surpass those of traditional PEG-modified regimens (Fig. 8C) [188].

In summary, multidimensional nanocarrier engineering innovations encompassing topological structural reinforcement, end-group chemical evolution, and surface coating substitution collectively establish a robust material foundation. This foundation enables nucleic acid therapeutics to successfully cross complex respiratory barriers.

### Antimicrobial agents and antibiotics

**Fig. 9:**
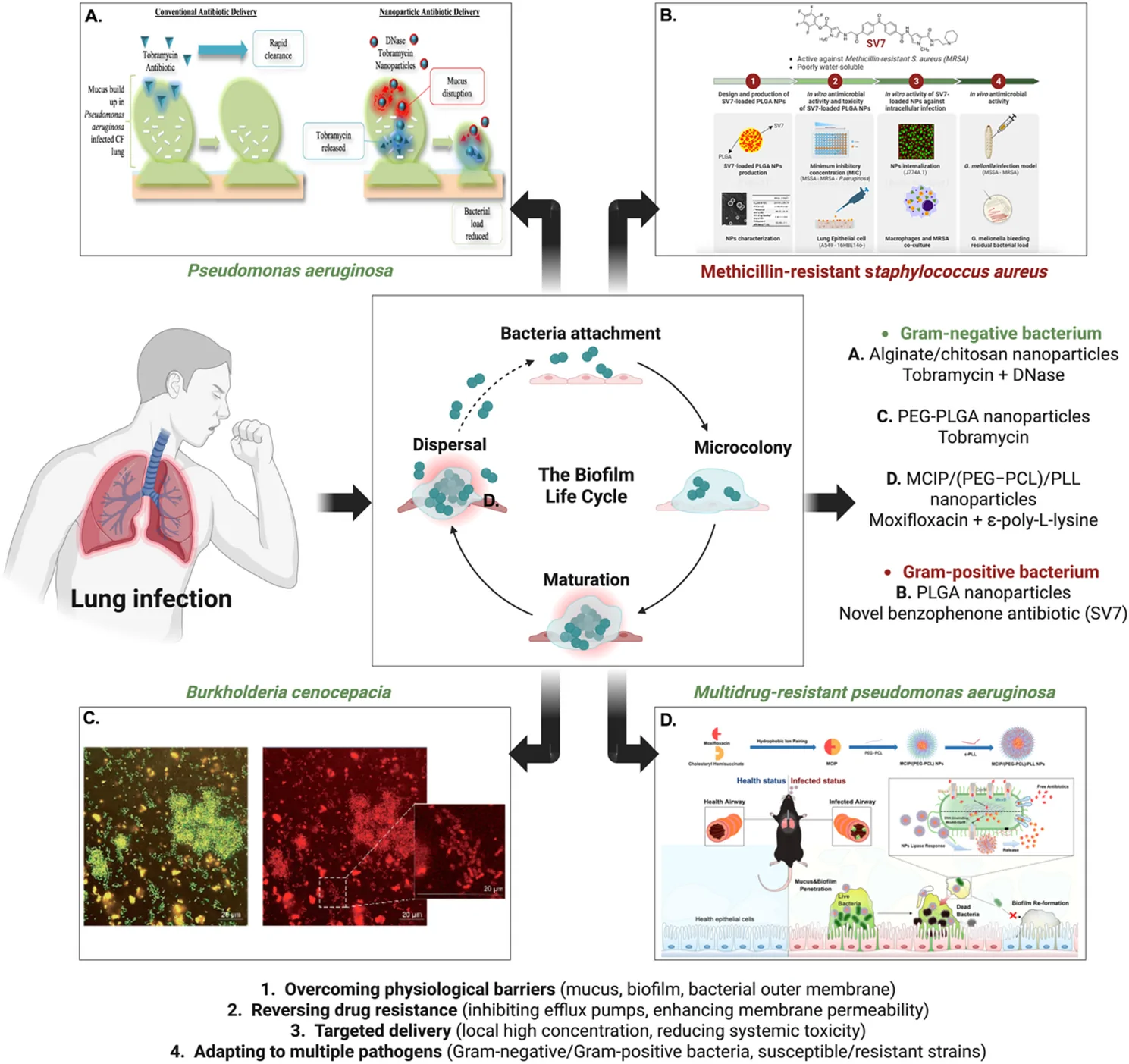
Schematic Illustration of Multifunctional Nano-antibiotic Delivery Platforms for Cystic Fibrosis-associated Chronic Bacterial Pulmonary Infections. This figure depicts the design of diverse polymeric nanoformulations targeting key respiratory pathogens: **(A) ***P. aeruginosa *[189], **(B)***B. cenocepacia *[190], **(C) ***MRSA *[191], **(D) ***MDR-p *[192]. aeruginosa, the bacterial biofilm life cycle in the lung, and four core therapeutic advantages of nano-antibiotics over conventional antibiotic therapy. Reproduced with permission

Although nucleic acid therapies address the foundational genetic etiology of chronic respiratory diseases, persistent bacterial infections constitute the predominant driver of patient morbidity and mortality. The clinical efficacy of conventional antibiotics is frequently compromised by formidable anatomical barriers and the rapid emergence of multidrug resistance. Consequently, nanomedicine has progressed beyond passive drug formulation to become an active therapeutic intervention. This section elucidates how advanced nanocarrier engineering effectively breaches pathological mucosal architectures, strategically reverses established bacterial resistance mechanisms, and harnesses the intrinsic antimicrobial properties of novel materials to fundamentally redefine pulmonary infection management (Fig. 9).

#### Overcoming the mucus and biofilm barriers

In chronic respiratory infections, the clinical failure of free antibiotics rarely originates solely from bacterial target mutations. Rather, these therapeutic agents are frequently hindered by the dual physical barriers composed of abnormal CF mucus and dense extracellular polymeric substance biofilms [193]. Consequently, the engineering design of nanocarriers must undergo a dialectical shift from passive drug encapsulation toward active barrier penetration and sustained release[194].

The nanoscale topological structure of the delivery vehicle directly dictates its capacity to permeate these physiological obstacles. In CF patient-derived sputum and CF airway epithelial air-liquid interface co-culture models, biomimetic lipid-liquid crystal nanoparticles leverage their unique internal mesostructures to forcefully drive tobramycin through dense mucoid *Pseudomonas aeruginosa* biofilms isolated from CF patients [195]. Within these clinically relevant CF models, nebulized tobramycin liquid crystal nanoparticles completely eradicated pathogens at concentrations reaching 1 × 10^9^ CFU/mL after merely two interventions. This bactericidal efficacy represents a 100-fold improvement over unformulated antibiotics. Furthermore, the nanostructure precisely restricts nonspecific drug permeation into the healthy pulmonary epithelial barrier to ensure an exceptional safety profile.

Beyond the optimization of physical morphology, the environmental adaptability of the surface chemistry remains central to achieving deep biofilm penetration. Ultra-small TPGS-PLGA hybrid nanoparticles establish a mucus-inert shell to successfully circumvent the nonspecific steric hindrance and charge entrapment typically imposed by mucins. Once these particles successfully descend into the deeper layers of the biofilm, the local bacterial microenvironment enzymatically strips away their outer shell. This biological trigger converts the solid polymeric core into a sustained release depot for azithromycin [196]. This precise intervention spans the dual dimensions of spatial permeation and temporal controlled release. It fundamentally resolves the pharmacokinetic deadlock characterized by inadequate deep-tissue concentrations of traditional antibiotics.

#### Resistance reversal and virulence microenvironment remodeling

In the face of multidrug-resistant (MDR) pathogens, the traditional monolithic strategy of direct bacterial eradication often exerts severe evolutionary selection pressure, thereby accelerating the screening and proliferation of resistant strains [197]. Consequently, the conceptual framework of antimicrobial nano-therapeutics has fundamentally evolved from passive bactericidal targeting to the active remodeling of the virulence microenvironment and the reversal of resistance mechanisms.

First, intercepting bacterial colonization at its source serves as the primary defense against the emergence of resistance. Innovative nanomaterial engineering confirms that phenylboronic acid-modified polyethyleneimine (PEI-BA) can specifically target airway mucin glycans through multivalent binding, effectively thwarting the initial adhesion of *Pseudomonas aeruginosa* via a strictly non-bactericidal physical competition mechanism. This microenvironment remodeling strategy avoids imposing survival selection pressure on the bacteria while generating significant synergistic efficacy when paired with conventional antibiotics like tobramycin [198].

Furthermore, cutting-edge nanocarriers can co-opt the pathogen’s own arsenal by converting secreted virulence factors into precise triggers for antibiotic release. For example, imipenem-loaded mesoporous silica nanoparticles coated with elastin and alginate lyase can specifically respond to the localized proteolytic activity of the pathogen [199]. While achieving precise controlled drug release, this virulence-responsive nano-system effectively degrades up to 80% of the biofilm matrix and induces a drastic three-log reduction in the viability of deep-seated bacteria.

Finally, combinatorial nano-delivery demonstrates the capacity to fundamentally reverse established transmembrane resistance mechanisms. Lipase-responsive polymeric nanoparticles encapsulating moxifloxacin and the antimicrobial peptide ε-poly-L-lysine (ε-PLL) not only forcefully penetrate dense biofilms but also significantly suppress the transcription of the MexAB-OprM efflux pump in MDR *P. aeruginosa*. Evaluated in rigorous in vivo infection models, this intervention successfully cleared 99% of the pulmonary pathogens [200]. This multidimensional nano-strategy integrates adhesion blockade, virulence responsiveness, and efflux pump inhibition to definitively strip superbugs of the survival advantages conferred by drug resistance.

#### Intrinsic antimicrobial properties of alternative nanomaterials

In the management of refractory respiratory infections such as CF, traditional strategies have frequently relegated nanoparticles to the status of inert drug delivery vehicles. As materials science advances, however, the conceptualization of cutting-edge nanocarriers has undergone a dialectical elevation. The delivery vehicles themselves, alongside non-traditional alternative antimicrobial payloads, are now demonstrating potent intrinsic pharmacological activities and anti-resistance capabilities.

To comprehensively bypass the inherent resistance mechanisms of conventional antibiotics, researchers have brought plant-derived natural antimicrobial molecules into the therapeutic landscape. Addressing the physicochemical drawbacks of poor aqueous solubility and high volatility associated with these compounds, investigators successfully fabricated polythioether nanoparticles using a solvent-free miniemulsion polymerization technique [201]. This methodology directly employs essential oil constituents like thymol and carvacrol as the reactive solvent. Consequently, this engineering approach eliminates tedious post-polymerization purification steps while achieving a remarkably high drug loading capacity of approximately 50% (w/w) and an encapsulation efficiency exceeding 95%. Importantly, this bactericidal efficacy was validated against clinical isolates of *Burkholderia cenocepacia* obtained from CF patients, demonstrating over 99.9% killing within 24 h. While these results are promising, it should be noted that the study was conducted in planktonic culture, and the efficacy of these nanoparticles against *Burkholderia cenocepacia* biofilms grown in CF sputum remains to be evaluated.

Concurrently, the intrinsic physicochemical architecture of the nanocarrier possesses an independent capacity to remodel the pathological microenvironment. Nanoparticles constructed from polyelectrolyte surfactants exemplify this phenomenon. Beyond functioning as highly effective delivery platforms for cationic antimicrobials, the polyelectrolyte surfactant formulation inherently exerts a profound mucolytic effect on artificial CF mucus models. Following nebulized inhalation, these nanoparticles exhibit dual intrinsic activities of physical intervention and cargo transport without inducing observable in vitro cytotoxicity. Furthermore, they achieved an impressive pulmonary retention rate of over 70% at 24 h in live murine models [202]. This avant-garde “carrier-as-drug” rationale opens a completely new approach for developing multi-targeted, synergistic inhalable antimicrobial therapies.

### Critical appraisal: benefits and limitations of cf nano-therapeutics

The preceding chapters have systematically elucidated how nanotechnology specifically addresses the three core bottlenecks of conventional CF therapy: inherent physicochemical deficits of existing drugs, complex multi-layered pathological biological barriers, and the unmet medical need for patients with rare mutations (Table 3). Nano-therapeutics have evolved from mere dosage form modifications into a core driving force reshaping the therapeutic model of CF, and their core value is manifested in three distinct dimensions: (1) Precise targeted delivery systematically reverses the pharmacokinetic defects of conventional drugs. Inhalable nanocarriers directly deliver CFTR modulators, antibiotics and biological agents to airway lesions, avoiding hepatic first-pass metabolism and systemic adverse effects, and reducing the equivalent therapeutic dose by several orders of magnitude. (2) Multi-target synergistic co-delivery achieves spatiotemporally controlled release of drugs with different mechanisms of action for the first time. This approach simultaneously addresses the three core pathological processes of CFTR dysfunction, mucus obstruction and bacterial infection, yielding synergistic effects that cannot be achieved through conventional free-drug combinations. (3) Breakthroughs in non-viral nanocarriers provide a viable disease-modifying therapeutic approach for approximately 10% of patients with rare mutations who cannot access small-molecule modulators. Notably, lung stem cell-targeted gene editing technologies have demonstrated the potential for lifelong cure with a single administration.

Nevertheless, a critical perspective is required to objectively evaluate the boundary of advantages and translational gaps of nano-therapeutics. The remarkable efficacy reported to date is predominantly derived from young animal models with mild disease and in vitro cell experiments, which fail to accurately reflect the highly heterogeneous pathological microenvironment in the lungs of human CF patients. Severe stenosis or complete obstruction of airways in end-stage patients prevents inhaled nanoparticles from reaching deep lesions. Substantial interindividual variations in mucus viscosity, charge density and biofilm architecture also cause the delivery efficiency of the same carrier to differ by more than one order of magnitude across patients. Additionally, nanotechnology has not fundamentally resolved the drug resistance problem posed by bacterial persister cells. Pathogens in a metabolically dormant state deep within biofilms can still cause recurrent infections after treatment cessation.

More critically, the clinical translation of nano-therapeutics extends far beyond biological efficacy validation. It faces systemic challenges in industrial manufacturing, regulatory science and long-term safety. These challenges are not secondary technical details but core bottlenecks that determine whether nanomedicines can move from the laboratory to clinical practice and benefit the broader patient population. The subsequent chapter will delve into these clinical translation barriers, analyze their underlying causes, and propose potential solutions to provide a clear roadmap for the future development of CF nano-therapeutics.

**Table 3:** Systematic Comparison of Advanced Nanocarrier Systems for Cystic Fibrosis Therapeutics

Therapeutic Class	Encapsulated Payload	Nanocarrier Formulation	Core Physicochemical Parameters	Mucus Penetration Mechanics and Key Outcomes	Stage of Research Progress
CFTR Modulator [156]	Lumacaftor & Ivacaftor	NLCs	Size: ~120 nm Zeta Potential: Near neutral EE: > 90%	Mechanism: Lipidic core bypasses the extreme hydrophobicity of modulators. Outcome: Simultaneously restores CFTR gating and trafficking with minimal systemic off-target toxicity.	Preclinical (CFTR-mutant in vitro models)
Mucolytic & Mucus Penetration [159]	NAC & Mannitol	Spray-dried Inhalable Microparticles	MMAD: 2–5 μm Moisture: < 5%	Mechanism: Deep alveolar deposition; Mannitol induces an osmotic gradient while NAC cleaves mucin disulfide bonds. Outcome: Synergistically hydrates the airway and eradicates pathological hyperviscosity.	Preclinical (Formulation optimization)
Gene Replacement [173]	CFTR mRNA	hPBAEs	Size: ~100–120 nm Zeta Potential: Highly positive (stable in aerosol)	Mechanism: Resists nebulization shear forces during inhalation. Outcome: Achieves 24.6% transfection in total lung epithelial cells; yields > 100 ng/g localized functional protein.	Preclinical (in vivo inhalation models)
Durable Gene Editing [180]	Cas9 mRNA & sgRNA	SORT LNPs	Size: ~80–90 nm Zeta Potential: Near neutral under physiological pH	Mechanism: Tuned ionizable lipids prevent premature macro-aggregation with extracellular DNA-actin networks. Outcome: > 70% editing in lung stem cells; durable CFTR correction for up to 660 days.	Preclinical (in vivo murine & patient-derived HBECs)
Gene Silencing [184]	siRNA	PLGA-DPPC	Size: ~160 nm Zeta Potential: PEG-modulated neutral	Mechanism: Dense PEGylation provides a hydrophilic, mucus-inert shell. Outcome: Evades steric entrapment in viscoelastic sputum, ensuring robust endosomal escape.	Preclinical (in vitro Air-Liquid Interface cultures)
Antimicrobial [195]	Tobramycin	LCNPs	Size: ~180–200 nm Zeta Potential: Negative (−18.6 mV)	Mechanism: Unique bicontinuous liquid crystalline mesostructure forces rapid internal matrix penetration. Outcome: Complete eradication of 109 CFU/mL *P. aeruginosa* (100-fold efficacy increase over free drug).	Preclinical (ex vivo CF clinical isolates on ALI)
Antimicrobial	Imipenem	Elastin & Alginate Lyase-coated Silica NPs	Size: ~100 nm Zeta Potential: Polyanionic exterior	Mechanism: Surface alginate lyase actively digests the mucoid biofilm capsule. Outcome: Pathogen-secreted elastase cleaves the coating, triggering precise, virulence-responsive antibiotic release.	Preclinical (in vitro mucoid biofilm models)

This table systematically collates the fundamental physicochemical parameters and functional outcomes of diverse nanocarrier architectures designed for cystic fibrosis therapeutics. The empirical data highlight the critical role of surface engineering, such as near-neutral Zeta potentials achieved via PEGylation, dynamic charge reversal, or the incorporation of active mucolytic agents, in actively overcoming the highly viscoelastic and electronegative pathological mucus barrier. Furthermore, specific aerodynamic metrics (MMAD) are reported for dry powder formulations to reflect the stringent requirements for deep pulmonary alveolar deposition. Abbreviations: ALI, air-liquid interface; Cas9, CRISPR-associated protein 9; CFTR, cystic fibrosis transmembrane conductance regulator; CFU, colony-forming unit; DPPC, dipalmitoylphosphatidylcholine; EE, encapsulation efficiency; HBECs, human bronchial epithelial cells; hPBAEs, hyperbranched poly(β-amino ester)s; LCNPs, lipid liquid crystal nanoparticles; LNPs, lipid nanoparticles; MMAD, mass median aerodynamic diameter; mRNA, messenger RNA; NAC, N-acetylcysteine; NLCs, nanostructured lipid carriers; NPs, nanoparticles; *P. aeruginosa*, *Pseudomonas aeruginosa*; PEG, polyethylene glycol; PLGA, poly(lactic-co-glycolic acid); sgRNA, single guide RNA; siRNA, small interfering RNA; SORT, selective organ targeting

## Challenges and opportunities in clinical translation

Transitioning nanomedicines from microscopic laboratory settings to real-world clinical applications in CF encounters formidable translational barriers. This progression requires surmounting multidimensional hurdles that extend far beyond theoretical therapeutic efficacy [203]. The critical evaluation of these technologies must strictly confront the realities of industrial manufacturing, regulatory science, clinical trial benchmarks, and comprehensive toxicological profiles. Future clinical breakthroughs depend heavily on a rigorous understanding of these specific limitations.

The clinical translation of nano-therapeutics remains deeply entangled in the paradox of manufacturing scalability and commercial viability. Scaling up from milligram-level laboratory preparation to kilogram-level industrial production introduces severe chemistry, manufacturing, and control challenges. These hurdles are particularly pronounced under strict Good Manufacturing Practice environments. Multicomponent systems, such as messenger RNA-loaded LNPs or polymer-lipid hybrid carriers, possess highly sensitive physicochemical architectures [204, 205]. During large-scale processing, any minute fluid shear stress or temperature fluctuation can precipitate the collapse of the spatial topological structure of the particles, leading to premature drug leakage or irreversible aggregation. Furthermore, traditional terminal sterilization methods utilizing high heat or irradiation routinely destroy these fragile nanoscale assemblies, forcing manufacturers to rely on complex and expensive aseptic processing techniques. To address these vulnerabilities, pharmaceutical developers must integrate microfluidic continuous manufacturing technologies during the earliest stages of formulation. This engineering approach seeks to substantially enhance batch-to-batch structural reproducibility. However, the exceedingly complex manufacturing processes paired with substantial research and development costs are creating profound health equity challenges. The prohibitive potential pricing of targeted nano-formulations threatens to exclude vulnerable patient populations in low- and middle-income countries from reaping these technological dividends. Establishing affordable manufacturing costs and scalable architectural designs must serve as central, non-negotiable constraints for the future commercialization of CF nanomedicines.

The structural complexity of advanced nanomedicines has far surpassed the conventional review boundaries of existing international regulatory frameworks. Current evaluation systems established by the Food and Drug Administration and the European Medicines Agency were originally tailored for traditional small molecules or biologically distinct macromolecules. Conversely, a nanomedicine constitutes a highly complex supramolecular assembly where the carrier material frequently possesses the dual pharmacological attributes of both a physical excipient and an active interventional agent. Major regulatory agencies currently lack standardized assessment guidelines addressing the in vivo three-dimensional structural degradation kinetics and the unique off-target distribution profiles of inhalable nanocarriers [206]. This relative lag in regulatory science significantly prolongs the duration of clinical trials and inflates the trial-and-error costs for innovative pharmaceutical enterprises. Regulatory bodies increasingly demand rigorous quality by design principles to ensure that critical quality attributes, such as particle size distribution and encapsulation efficiency, remain tightly controlled across all clinical phases. The lack of distinct regulatory pathways for generic nanomedicines further complicates the eventual market entry of more affordable therapeutic alternatives. Despite these regulatory bottlenecks, early clinical trials provide vital benchmarks and expose the true severity of human biological barriers. For instance, the clinical evaluation of MRT5005, an inhalable mRNA LNPs formulation developed to restore CF transmembrane conductance regulator function, offered important clinical proof-of-concept [207]. However, the trial simultaneously exposed the profound difficulty of translating high transfection efficiencies observed in preclinical murine models directly to the thickened, hyper-inflammatory mucus environment of the human airway. Contemporary candidates like VX-522, an mRNA therapy currently navigating active clinical trials, and SPL84, an inhalable antisense oligonucleotide, are actively confronting these exact biological discrepancies. These real-world translational efforts underscore the absolute necessity of evaluating nanomedicines in highly biomimetic, mucus-competent human airway models prior to initiating formal regulatory submissions.

While the targeted delivery capabilities of nanomedicine offer substantial clinical benefits by protecting fragile payloads and enhancing cellular uptake, addressing the inherent toxicological risks is absolutely essential. Chronic respiratory interventions require frequent, lifelong administration, which profoundly amplifies the potential for cumulative harm. A balanced evaluation of therapeutic efficacy against potential cellular and systemic toxicity must drive all formulation optimization. Cytotoxicity remains a primary concern for non-viral vectors. To penetrate the dense sputum barrier and achieve cellular endocytosis, nanocarriers frequently employ cationic polymers or ionizable lipids. These highly charged components can interact unselectively with the anionic phospholipid bilayers of healthy endogenous cells. Such interactions disrupt cell membrane integrity and induce dose-dependent cytotoxicity within the respiratory epithelium. Researchers must meticulously titrate the surface charge density to maintain a delicate balance between sufficient cellular uptake and acceptable cellular viability. The clinical benefit of achieving robust gene correction must not come at the cost of widespread epithelial necrosis. Immunogenicity presents an equally formidable challenge. Nanocarriers typically require intensive surface chemical modifications, such as PEGylation, to evade rapid mucosal clearance and macrophage phagocytosis. For CF patients who require repeated dosing over decades, these exogenous artificial modifications easily induce the accelerated blood clearance phenomenon mediated by anti-PEG antibodies. Furthermore, repeated pulmonary exposure to synthetic lipid or polymeric components may provoke unpredictable local immune cascades. This foreign body response threatens to exacerbate the baseline hyper-inflammatory state already dominating the pathological airway, potentially accelerating lung function decline. Finally, the long-term safety of chronic nanoparticle inhalation raises critical bioethical and physiological concerns. Synthetic nanomaterials frequently possess incomplete biodegradability, creating significant risks regarding accumulation toxicity in deep lung tissues and alveolar macrophages. This is especially alarming for pediatric patients who are in critical stages of pulmonary and systemic organ development. Whether prolonged respiratory exposure to specific nanoscale materials will trigger unknown transgenerational epigenetic modifications or unforeseen developmental disruptions remains an open clinical question. The definitive clinical advantage of utilizing nanomedicine lies in its unparalleled ability to rewrite the genetic basis of CF. However, to secure this benefit safely, future therapeutic designs must prioritize the development of fully biodegradable and inherently low-immunogenicity materials to fundamentally circumvent the long-term toxicity risks associated with advanced synthetic components [208].

## Conclusion and future directions

Clinical translation of CF-targeted nanotechnology requires distinct differentiation between analytical verification and scalable clinical qualification. Among emerging diagnostic tools, epidermal microfluidic devices with colorimetric sweat-test assays are the most mature for point-of-care clinical use. These systems have advanced beyond preclinical testing to preliminary patient cohort validation, outperforming electrochemical genosensors and volatile organic compound sensors, which are still limited to testing in simulated biofluids. For therapeutics, mRNA LNPs and mucus-penetrating polymer carriers show the greatest potential for near-term clinical advancement. Their proven structural stability has enabled early-phase human trials, making them suitable for treating patients carrying rare or nonsense mutations unsuitable for standard small-molecule CFTR modulators.

Despite these preclinical advances, chemistry, manufacturing and controls bottlenecks hinder broad clinical adoption. Large-scale good manufacturing practice-compliant manufacturing of complex multicomponent nanocarriers frequently impairs particle integrity, causing unstable drug encapsulation, poor inhalation aerodynamic properties or accelerated in vivo clearance. To narrow this translational divide, future preclinical research needs standardized validation criteria. Researchers should move past overreliance on generic acute rodent pulmonary infection models and instead validate formulations using CF-relevant substrates: patient-isolated sputum, human airway epithelial air-liquid interface cultures and CFTR-knockout animal models. Such tailored testing improves the predictability of preclinical findings for human clinical performance.

## Data Availability

No datasets were generated or analysed during the current study.

## Abbreviations

ΔF508: Deletion of phenylalanine at position 508
ASO: Antisense Oligonucleotide
ATP: Adenosine Triphosphate
AuNPs: Gold Nanoparticles
CF: Cystic Fibrosis
CFTR: Cystic Fibrosis Transmembrane Conductance Regulator
CRISPR: Clustered Regularly Interspaced Short Palindromic Repeats
IL-6: Interleukin 6
IL-8: Interleukin 8
IV: Intravenous
LNP: Lipid Nanoparticle
MDR: Multidrug-Resistant
mRNA: Messenger Ribonucleic Acid
NAC: N-acetylcysteine
NF-κB: Nuclear Factor-kappa B
PEG: Polyethylene Glycol
pDNA: Plasmid DNA
QbD: Quality by Design
RNA: Ribonucleic Acid
rhDNase I: Recombinant Human Deoxyribonuclease I
siRNA: Small Interfering RNA
